# A Finger-Based Numerical Training Failed to Improve Arithmetic Skills in Kindergarten Children Beyond Effects of an Active Non-numerical Control Training

**DOI:** 10.3389/fpsyg.2020.00529

**Published:** 2020-03-24

**Authors:** Ulrike Schild, Anne Bauch, Hans-Christoph Nuerk

**Affiliations:** Department of Psychology, University of Tübingen, Tübingen, Germany

**Keywords:** finger-number associations, initial arithmetic skills, embodiment, intervention, children

## Abstract

It is widely accepted that finger and number representations are associated: many correlations (including longitudinal ones) between finger gnosis/counting and numerical/arithmetical abilities have been reported. However, such correlations do not necessarily imply causal influence of early finger-number training; even in longitudinal designs, mediating variables may be underlying such correlations. Therefore, we investigated whether there may be a causal relation by means of an extensive experimental intervention in which the impact of finger-number training on initial arithmetic skills was tested in kindergarteners to see whether they benefit from the intervention even before they start formal schooling. The experimental group received 50 training sessions altogether for 10 weeks on a daily basis. A control group received phonology training of a similar duration and intensity. All children improved in the arithmetic tasks. To our surprise and contrary to most accounts in the literature, the improvement shown by the experimental training group was not superior to that of the active control group. We discuss conceptual and methodological reasons why the finger-number training employed in this study did not increase the initial arithmetic skills beyond the unspecific effects of the control intervention.

## Introduction

Being able to competently deal with numbers is a fundamental skill in our society. Recently, the interest of researchers has turned to precursor abilities of mathematical achievement like approximate number processing (for a review see De Smedt et al., 2013; Libertus et al., 2016), spatial skills (e.g., Cipora et al., 2015), spatial number associations (e.g., Cipora et al., 2015), verbal number skills (e.g., Libertus et al., 2016), counting (e.g., Nguyen et al., 2016), mathematical language (Purpura et al., 2017) or base-10-knowledge (Moeller et al., 2011). Another of these potential precursors might be finger representation or finger gnosis (see Moeller and Nuerk, 2012 for a discussion). In turn, finger gnosis may serve to build up associations between fingers and numbers. It has been argued that finger representations might be another important precursor for initial arithmetic skills as they provide the child with an embodied representation of numbers developmentally located at the transition between early non-verbal representations and cultural symbolic representations. Such arguments rest on theoretical considerations (e.g., Moeller et al., 2011; Moeller and Nuerk, 2012) and observed correlations; however, whether earlier finger-number relations really have effects on later arithmetic skills has rarely been investigated. Therefore, the core purpose of this study was to examine intervention effects of finger-number associations on early arithmetic skills.

There is solid evidence now that finger and number representation are associated. First evidence was provided by Gerstmann (1940) who described neurological syndromes like finger agnosia, agraphia, acalculia and a disorientation for right and left that occurred together. This combination of deficits suggests that the same brain regions are responsible for the underlying processes. Over the last decades, studies using brain imaging techniques supported this close connection. Overlapping brain regions were found for finger representations and brain areas involved in number counting (e.g., Tschentscher et al., 2012) or arithmetic calculations (e.g., Berteletti and Booth, 2015). Many behavioral studies in adults also support an association of, for example, finger representation and counting [but see Brozzoli et al. (2008) for a dominance of a mental-number line representation when directly contrasted with finger-number representations], of finger representation and cardinality, and of finger representation and arithmetic (for a short overview see Di Luca and Pesenti, 2011). However for behavioral, as well as for brain imaging studies, most evidence so far is correlational – a truly *causal* relation between finger representation and numerical/arithmetic skills by manipulating finger knowledge and built-up representations has rarely been shown. Whether children refine their finger representations in parallel or in mutual interaction with the acquisition of their initial numerical skills or whether a good finger representation is beneficial or even necessary for developing numerical representations and/or numerical competencies is an open and controversial question in numerical development and education (Moeller and Nuerk, 2012).

A growing number of studies showed that finger representation (or finger gnosis) is associated with basic numerical skills (Costa et al., 2011) and that finger gnosis can predict later numerical skills (Fayol et al., 1998; Noël, 2005). However, the explained variance tends to be small. This was particularly the case when possible third variables like general cognitive ability were taken into account, and a sufficient number of participants was tested (Penner-Wilger et al., 2007, 2009; Kohn et al., 2015; Poltz et al., 2015; Wyschkon et al., 2015; Long et al., 2016; Wasner et al., 2016). Nonetheless, finger representations do seem to affect numerical processing in both children and adults as shown, for example, in the finger-based sub-base five effects (e.g., Domahs et al., 2008, 2010). To additionally investigate the role of finger gnosis as a precursor for later arithmetic skills, a sub-purpose of our study was to look at the predictive value of finger gnosis at pre-intervention for initial mathematical skills at post-intervention.

However, it is important to distinguish between finger gnosis or finger representations, finger-number associations and direct finger use in finger counting and arithmetic tasks.

Concerning finger use in number tasks, when children start to communicate about numbers or when they learn to count, they often use their fingers (e.g., showing their age with their fingers). This is even true for blind children (Crollen et al., 2011a; but see Crollen et al., 2014 for the role of visual experience in finger-number associations) or for children without hands who use their phantom fingers to count (Poeck, 1964). Even later when starting to acquire addition and subtraction skills many children use their fingers (e.g., Butterworth, 1999). Furthermore, when prevented from using their fingers by interfering hand movements arithmetic performance seems to drop (Crollen and Noël, 2015). This shows that fingers are used in a numerical and arithmetic context but does not imply that this finger-number association leads to better arithmetic performance.

Children who use their fingers directly might have ‘good’ finger representations and finger-number associations. In contrast, children who do not use their fingers directly, might have either ‘poor’ finger representation and in turn ‘poor’ finger-number associations, which prevents them from using their fingers. Or they might have ‘very good and stable’ finger representations and finger-number associations, but are no longer in need of using their fingers directly, because they have already built up good abstract numerical representations. Thus, conclusions about the relation between direct finger use and underlying (finger or numerical) representations should be drawn with caution. This would also be in line with the results of Lafay et al. (2013) who showed that with 4–7 year-olds finger gnosis was related to an enumeration task, but not to direct finger use in counting. In this context, Reeve and Humberstone (2011) have identified four subgroups of 5–7 year-old children based on their performance on an addition task and spontaneous finger use. In this classification, high performers rarely used their fingers directly, whereas moderate performers belonged to one of two groups: either to a group with high, or to a group with low, direct finger use. Finally, their fourth group contained low performance children and low finger use. In addition, Wasner et al. (2015) have shown for adults that the use of specific fingers can vary according to the demanded underlying principle of the task (e.g., requiring either ordinality or cardinality or 1-1 relations). This indicates that finger use is highly flexible and also depends on the task itself. Yet, training of finger gnosis and direct finger use in numerical tasks might have a double advantage for children. First, it may improve finger gnosis and finger representation itself. Second, it may help children to grasp the abstract format of numbers by using an embodied format of numbers (Moeller et al., 2012).

If numerical skills were rooted in finger representations, one would assume a universally applicable sequential development from using numerical gestures first to using abstract verbal numbers second. Piaget (1954) claimed that abstract concepts emerge from senso-motoric experiences. A study by Nicoladis et al. (2010) calls such a sequential development into question. They showed that preschoolers were actually better at processing number words than at processing number gestures. Thus, at least for counting, they did not find number gestures to precede the use of symbolic number words. In a similar vein, Crollen et al. (2011a) have shown that blind and sighted 7–13 year-old children performed similarly in enumeration tasks despite less finger counting and more inconsistent finger-number associations on the part of the blind children (Crollen et al., 2011b). While both groups had equal finger discrimination abilities, blind children showed better working memory performance than sighted children. Thus, if finger counting facilitates the development of numerical skills in sighted children, then blind children might compensate for this effect with their superior working memory skills. This does not mean that finger counting cannot be useful (e.g., Lafay et al., 2013), especially for more complex and difficult tasks where finger counting could, for example, help to reduce working memory load (see also Crollen et al., 2011b). These studies suggest that although finger counting can be beneficial, it may not be necessary for developing counting abilities.

Intervention studies seem to be a promising tool to investigate whether there is a causal relation between finger gnosis, finger-number associations and arithmetic skills. Even though an increasing number of intervention studies have compared the contributions of potential precursor abilities for mathematic proficiency over the last years, only very few studies looked at the role of finger gnosis or finger-number association. To date, only a small number of studies have carried out finger-number trainings with school-aged children. For example, Gracia-Bafalluy and Noël (2008) provided a 30-min finger gnosis training session once a week, for an 8-week period, to first graders. The training was a ‘pure’ finger gnosis intervention designed to improve sensitivity and mobility of the fingers (e.g., labyrinth game or piano game). They observed that children with an initial poor finger gnosis benefited from the training and scored higher not only in finger gnosis, but also in numerical skills after the training. Unfortunately, their methodological procedure was rightfully criticized, because the authors did not consider the regression to the mean, which alternatively could explain the results (Fischer, 2010). In a recent study, Jay and Betenson (2017) trained 137 first graders in eight 30-min sessions during 4 weeks. The group playing finger gnosis games improved merely in the finger gnosis task. This is surprising, because in contrast to Gracia-Bafalluy and Noël (2008) their finger gnosis training involved not only ‘pure’ finger gnosis interventions, but also training in the cardinal and ordinal properties of numbers: Children actively verbalized numbers in games like finger counting, showing fingers-to-numbers or showing calculations with fingers. The group playing number games (e.g., domino, snake and ladders, playing with cards and dice) improved only in a non-symbolic magnitude comparison task. Finally, the third group, which had received a combination of both trainings, improved in their quantitative skills. The authors concluded that in the combined training children built up connections between different representations of numbers (e.g., finger-number, symbolic and non-symbolic representations), which might have led to the increased performance in quantitative skills compared to both single training groups.

Going beyond these two intervention studies Frey et al. (unpublished) trained 119 first graders not only in finger gnosis and finger counting, but also in using their fingers in arithmetic tasks in 18 sessions of approximately 25 min. Frey et al. (unpublished) trained the following skills: *Finger gnosis* was trained in the beginning of the intervention by differentiation and naming of the fingers, finger-thumb tapping and by tracing ways through labyrinths where children used each finger separately for finding different ways through various labyrinths. Further, children traced Arabic digits from 1 to 10 with their respective fingers or thumbs. *Ordinal number-finger association* was trained by a task asking children to count their fingers forward and backward thereby relating numbers to the respective finger. *Cardinal finger-number association* was trained, for example, by detecting numbers in a story. Here children had to indicate the numbers by showing their fingers. Further, they also played a memory card game with symbolic cards (digits), non-symbolic cards (points) and finger pattern cards featuring the numbers 1-9. Finally, most of the intervention games (nine tasks) trained *number relations* through the practice of addition and subtraction tasks while using the fingers (for a more detailed description of tasks see Frey, 2017). The results showed that trained children outperformed children of a control group in tasks including addition and subtraction up to a number range of 20, but not in number line estimation on a 0-to-50 and a 0-to-100 scale. Furthermore, these effects were still observed after 9 months. This study supports the view that training finger use in and beyond arithmetic tasks facilitates the learning of specific arithmetic skills. This does not necessarily mean that direct finger use while calculating increases the performance, but rather that the strengthening of the association between finger and number representations may lead to this improvement.

In sum, former studies have shown that primary school children improve in their arithmetic skills by finger-number training. However, some correlational studies suggest that finger-number relations might be predictors of later numerical skills and arithmetic already in preschoolers (Fischer et al., 2017; Suggate et al., 2017).

The aim of the present study is to investigate whether kindergarten children can profit from finger-number training, even before they receive formal math education in addition and subtraction. Training of other potential precursors has already been done (e.g., with non-symbolic approximate number training, Park et al., 2016; but see Szücs and Myers, 2017 for a critical review), but not with finger-number associations, to our knowledge. We are interested as to whether training finger-number associations in kindergarteners may pave the way for better future arithmetic skills as the training of phonological awareness paves the way for better future reading skills (e.g., Schneider et al., 1997; Bus and van Uzendoorn, 1999; Lundberg, 2009). To infer such a causal relation, it is important to train children before they receive formal instruction. For reading acquisition, this has been a debate for years: In school, literacy acquisition interacts with the acquisition of phonological awareness. Therefore, no clear conclusions about a causal relation can be drawn from children that already attend school (Castles and Coltheart, 2004). The same may also apply for the finger-number-arithmetic-relation examined here. The development of finger-number associations might interact with the acquisition of arithmetic proficiency.

To investigate whether finger-number associations can be trained in kindergarteners and whether this training affects arithmetic skills, we adapted the training of Frey et al. (unpublished) for kindergarten children aged five to six. An advantage in training younger children might be that for them finger representations might not be as mature as in older children. The same is true for finger-number associations, which may be less stable compared to older children. For that reason, both – finger representations and finger-number associations – might be even more susceptible to external training in younger compared to older children. That younger children might benefit more from interventions than older children has also been shown in other training studies with preschoolers (e.g., Park et al., 2016). In sum, we hypothesized that finger-number associations are causally related to numerical skills. If this is the case, then training of finger-number associations, especially in kindergarteners, may directly impact upon initial arithmetic performance – even before the beginning of formal arithmetic instruction and this impact should be larger than in a control group.

Although evidence for an influence on finger gnosis on later arithmetical performance seems rather small – if it exists at all – we incorporated some tasks of finger gnosis in the training, because finger gnosis seems to be necessary (but not sufficient) to associate fingers and numbers. In other words, if a child is not able to select or move a certain finger at all, they will also not be able to select this finger in associations with certain ordinal, cardinal or 1-1-finger-number relations. Thus, most tasks involving the assessment of active finger-number relation require some knowledge (i.e., here gnosis), of which fingers are to be involved in the task. Therefore, as a sub-hypothesis, we also wished to examine the question of whether finger gnosis at pre-test predicts initial mathematical skills at post-test.

However, it was not the aim of our study to show that training finger gnosis alone and unrelated to any finger-number relations has an effect on later arithmetic performance. We know that relations between finger gnosis and numerical skills are small to non-existent and have repeatedly argued (e.g., Domahs et al., 2010; Moeller et al., 2012) that the embodied representation of numbers with fingers, and not just finger gnosis alone, is essential.

Therefore, the core training feature concentrates on the finger-number associations as a precursor skill that might affect later arithmetic skills. However, we also include some early number relation tasks (completion to 5 and to 10) that may be on the border between finger-number associations and arithmetic skills (see section “Materials and Methods” for further details). Arithmetic knowledge of addition and subtraction were not directly trained, but they were accessed after the intervention. On purpose we decided to avoid training to the task because we wanted to investigate how the precursor skills of finger-number associations affect arithmetic skills without training arithmetical tasks by themselves.

In sum, the aim of this study was to investigate whether training finger-number associations in kindergarten improves initial arithmetic skills in elementary school. To the best of our knowledge, this is the first study that tries to show this causal relation by applying an intervention at kindergarten age with an active control group.

Here we wished to examine – as a first step – whether finger-number relations constitute a precursor of arithmetic skills, after taking into account an established predictor of early mathematical skills namely children’s non-verbal intelligence (e.g., Aragón et al., 2016). In addition, we included gender as it is a debated popular predictor. In several studies gender differences have been observed in some spatial representations of number (e.g., Bull et al., 2013; Reinert et al., 2017), in children’s early arithmetic skill (Krinzinger et al., 2012; Hornburg et al., 2017; see also Brunner et al., 2011), and even in adults’ arithmetic and numerical skills (Pletzer et al., 2013, 2016). However, many recent studies have not found that females and males differ, for example, in a meta-analysis of math performance (Hyde, 2016), in several studies on children at various stages of their development (Morsanyi et al., 2018; Bakker et al., 2019; Hutchison et al., 2019); and in an adult online study testing the SNARC effect with over 1000 participants (see supplementary materials of Cipora et al., 2019). Because of these diverging results in the literature, which may differ depending on task, sample, culture and paradigm, we included gender as a predictor to examine whether it has any effect on embodied learning of basic numerical skills.

In sum, finger-number relations that were systematically targeting different constructs (finger gnosis, 1-1 finger-number mapping, ordinality, cardinality, base-10, place-value knowledge) were trained to increase salience of the training. If such a training in kindergarten were successful, future studies could investigate – in a second step – which components of finger-number relations might contribute the most to this training effect As a third step, further research can then compare or combine such a finger-number training with other effective interventions that train other components of numerical knowledge to unravel differential effects of the various potential trainings.

## Materials and Methods

### Procedure

Preschool children received either finger-number training or one of two phonological control trainings. These phonological trainings belong to a training study on its own, but served as control training in the present study. The trainings were pseudorandomly assigned to local kindergartens to ensure that each training group comprised a similar number of children. For economical reasons, all children within the same kindergarten received the same training (but we tested whether there were pre-training differences between the kindergartens in the different experimental groups, which was not the case; see below). We allowed bilingual children to take part in the training, but only monolingual children were included in the study. Because our children were younger than the children in the study by Frey et al. (unpublished), we adapted the training’s extent and content to suit kindergarteners. Each training session was only approximately 10 min, but the training took place every day, for a period of 10 weeks (from February/March to May/June during the children’s final kindergarten year). Thus, the overall time of the training was nearly equal between our training study and that of Frey et al. (unpublished). In sum, we trained 18 groups of varying size (with a minimum of 4 children and a maximum of 10 children in the finger-number training). The training was conducted by instructed undergraduate students and doctorate members of the department of psychology of the University of Tübingen and took place in the kindergartens. Before and after the training we assessed each child’s arithmetic and language skills in one or two test sessions lasting between 30 and 60 min. Tests that were important for the actual study included measures of finger gnosis, addition, subtraction and completion to 5/10. We also administered tests that were language specific to evaluate the phonological training. The results of the language study will be reported elsewhere.

### Participants

In total 102 children took part in the training, and contributed data to both pre- and post-tests. The experimental group consisted of 35 children who received the finger-number training. The control group consisted of 67 children who received either the phonological training (*N* = 37, 23 male) or the phonological-orthographic training (*N* = 30, 17 male) as control trainings (see Table 1 for demographic data)^1^. Participants received a present for each test session. Both children and their parents gave their informed consent. All children who took part in the tests were monolingual native speakers of German.

**TABLE 1:** Demographic data and differences between groups in age, sex, attended days, handedness measured by the Lateralized Quotient (LQ; Oldfield, 1971) and in the subtest Matrices taken from the Culture Fair Intelligence Test (CFT 1-R; Weiß and Osterland, 2013).

	Age to pre-test [years; month (range)]	Sex (male/female)	Attended days [mean (SE, range)]	Handedness LG [mean (SE)]	Subtest Matrices [mean (SE)]
Experimental group	5.10 (53–6.11)	19/16	41.2 (1.15, 23.5–0)	70 (7.06)	5.8 (0.63)
Control group	5.11 (5.2–7)	40/27	40.0 (0.90, 7–49)	56 (6.54)	6.8 (0.42)
Significant differences between groups	*t* < 1, *ns*	**χ2 < 1, ns**	*t* < 1, *ns*	*t* = 1.3, *p* = 0.154, *ns*	*t* = 1.3, *p* = 0.192, *ns*

### Materials and Tests

#### Training Material

The training material was adapted from Frey et al. (unpublished), and consisted of 18 different short games in total. We trained the following skills: *Finger gnosis* contained tasks like finger tapping and tracing a way through a labyrinth with specific fingers. Note, that these two tasks did not involve numbers. *1-to-1 mapping* of fingers and numbers included naming the fingers and mapping numbers to single fingers; learning Arabic digits was covered by tracing a number on a sheet with the respective fingers. *Ordinal finger-number associations* were trained by finger counting in various games (e.g., finger counting, object counting and counting of claps) and by ordering numbers, for example, by placing numbers in the right order and ordering a deck of cards displaying fingers, digits and points. The training of *cardinal finger-number associations* included games like naming the number corresponding to fingers presented, detecting numbers that were hidden in stories, playing a memory card game with cards displaying fingers and numbers, playing a bingo game with cards displaying fingers and sheets displaying numbers and playing a domino game with cards displaying fingers and numbers. Finally, *number relations* in *the base-10 and place-value system* and finger-number mapping were trained by completion of 5/10 tasks (one with fingers and one with a deck of cards displaying numbers) and by doubling numbers (showing double the number of fingers shown by the trainer). All games include the use of the fingers. In each training session up to three games were played depending on the length of the games (to see how often each game was played and for further details please refer to Appendix Table A1). The idea of having so many different games was not only to train different conceptual levels with increasing difficulty, but with 50 sessions it is also essential to vary the games to keep the children interested and motivated. The control training included phonological games of similar duration.

#### Pre- and Post-tests

##### Handedness

We used the lateralized quotient (LQ) of the Edinburgh inventory (Oldfield, 1971) to assess handedness, but we left out the item ‘Striking Match.’

##### Finger gnosis

We used the same finger gnosis assessment as in Wasner et al. (2016) who adapted a task and procedure previously used by Noël (2005), Gracia-Bafalluy and Noël (2008), and Reeve and Humberstone (2011). For the first task, a box was placed over the hand of the child. The trainer touched a single finger on the middle phalanges and asked the child to show the tapped finger. This was done with both hands, respectively (maximum 6 points, 3 points for each hand). Thereafter, two fingers of one hand were touched consecutively. The child earned one point for each correct finger and another point for the correct order (maximum 20 points, 10 points for each hand). In the second task both hands were placed behind the box. Two pictures of the right and left hand were placed beside the box. The trainer touched one finger of the child and one finger of the picture at the same time. The child indicated whether the fingers were the same or not (4 points). Finally, children solved the same task, but with two fingers in succession (4 points). The maximum number of points was 34.

##### Completion-to-5/10

We introduced the completion-to-5 test with the following example: “Now I want you to tell me how many gummy bears we need to reach 5. If I have 4 gummy bears, how many more gummy bears do I need to reach 5?” A similar instruction served for the completion-to-10 task. The test stopped after 3 min. At pre-test, the maximum number of points was 15, and at post-test the maximum number of points was 30.

##### Addition

First, we familiarized the children with the concept of addition. At pretesting, children solved at maximum 25 tasks in the number range from 1 to 10. During post-testing, a maximum of 35 problems were presented (here the single numbers of the last 10 tasks ranged between 10 and 20). Children had 4 min to solve as many tasks as possible.

##### Subtraction

Again, we first familiarized the children with the concept of subtraction. At pretesting, children solved a maximum of 20 subtraction tasks in the number range of 1–10. At post-testing there were 30 problems. Thus, the maximum number of points was 30. Here, the numbers for the last five tasks ranged between 10 and 20. Again, the test stopped after 4 min.

##### General cognitive abilities

For a measure of general cognitive abilities, we administered two subtests (*Matrices* and *Continuing Rows*) of the Culture Fair Intelligence Test (CFT 1-R; Weiß and Osterland, 2013) at post-test. However, as various trainers reported that children had difficulties with the Continuing Rows subtest, we only entered the Matrices subtest into analyses.

All of the tasks were presented orally to the children and required a verbal response except the two tasks measuring general cognitive abilities where visual material was used in addition.

## Results

**FIGURE 1:**
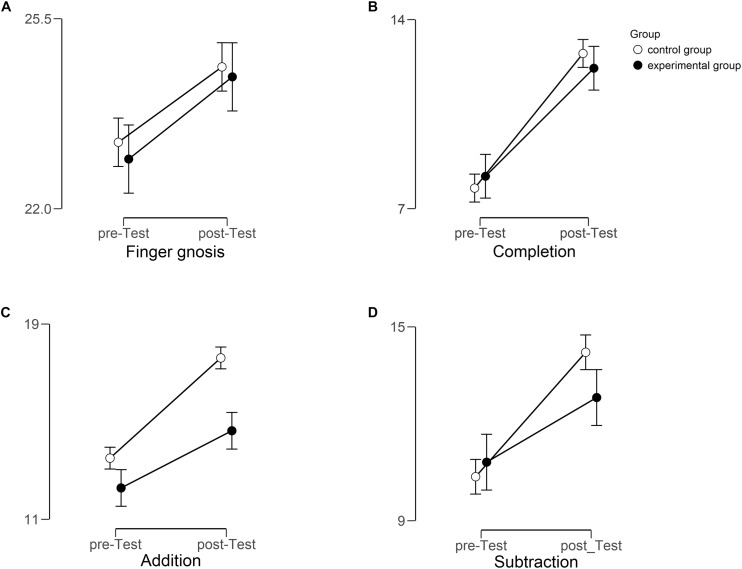
Mean scores for all dependent variables [**(A)** Finger gnosis, **(B)** Completion, **(C)** Addition, and **(D)** Subtraction] for each group (experimental group [black] versus control group [white]) and each time (pre- versus post-test). Error bars indicate standard errors. Note, that the figure for subtraction displays results of the reduced sample (*N* = 63, for more details refer to the text).

Each dependent measure (finger gnosis, completion, addition, subtraction) was subjected to a repeated measures ANOVA with the within-factor *Time* (pre-test versus post-test) and the two between-factors *Group* (experimental group versus control group) and *Sex* (male versus female) together with the co-variate *CFT-matrices*. The scores of the *CFT-matrices* were centered. Figure 1 displays the mean scores of each dependent variable separately for each group and pre- and post-tests, respectively.

Independent *t*-tests showed that there was no hint of pre-test differences between experimental and control group for all tasks, *t*_all_ ≤ 0.731, *p* ≥ 0.466. All dependent measures showed that improvement took place over time implicating that the measures we used were sensitive to intra-individual changes.

### Finger Gnosis

The ANOVA revealed a main effect of *Time, F*(1,97) = 5.911, *p* = 0.017, η*^2^* = 0.056. The co-variate *CFT-matrices* was also significant, *F*(1,97) = 9.357, *p* = 0.003, η*^2^* = 0.088. No other main effects or interactions were significant.

In order to quantify the null-effect of the interaction of interest (*Time* and *Group*) we applied Bayesian repeated measures ANOVA as implemented in JASP-software (JASP Team, 2017, Version 0.8.2). To get more assurance about the probability of the null hypothesis, we decided to run a Bayesian analysis. However, as there is no golden standard available, especially for repeated measures with within and between factors, we opted for the most simple and comprehensible way. We excluded Sex and CFT-matrices from the Bayesian analysis, because Sex was of no special interest here (and similarly distributed between groups) and CFT-matrices did not significantly differ between groups (see Table 1). We treated all main factors as nuisance factors to find out whether the interaction of interest (Time and Group) showed a higher probability for the null model or for the alternative model or whether it lay in between both models. The Bayes factor B01 indicates how much better the data predicts the null hypothesis compared to the alternative model. The detailed results of these analyses are provided in the Supplementary Table S1. For finger gnosis we set up a null model by excluding *CFT-matrices* and *Sex* and including each of the main factors (*Time* and *Group*) as nuisance variables. We compared this null model with an alternative model that included the interaction of interest (*Time* and *Group*). The model comparison revealed a BF_01_ of 5.06 for the interaction and a probability of *p*(H_0_| D) = 0.83 which is substantial/positive evidence (Jarosz and Wiley, 2014) for the null model (see Supplementary Material for tables with Bayes Factors).

Each dependent post-measure (Finger gnosis, Completion, Addition and Subtraction) was additionally submitted to an ANCOVA with the fixed factors Group and Sex and the co-variates CFT-matrices and the respective pre-measure. Results of all ANCOVAs were nearly identical to the results of the ANOVAs (see Supplementary Table S2 for detailed information).

Similar to the Bayesian repeated measures ANOVA we also ran Bayesian ANCOVAs. Here, we set up the null model by excluding CFT-matrices and Sex and by including the fixed factor Group. The co-variate Pre-measure was treated as a nuisance variable. We compared the null model with the alternative model that included the main effect of interest, namely Group. Results of Bayesian ANCOVAs were nearly identical to the Bayesian ANOVAs (see Supplementary Table S3).

### Completion-to-5/10

The ANOVA revealed a main effect of *Time, F*(1,97) = 47.616, *p* < 0.001, η*^2^* = 0.316 and an effect of *CFT-matrices*, *F*(1,97) = 23.105, *p* < 0.001, η*^2^* = 0.190, and an interaction of both of these factors, *F*(1,97) = 5.259, *p* = 0.024, η*^2^* = 0.035. No other main effects or interactions were significant. Comparing the null model (excluding *CFT-matrices* and *Sex* and including the main factors *Time* and *Group* as nuisance variables) with the alternative model (including the interaction of *Time* and *Group)* revealed a BF_01_ of 3.56 for the interaction and a probability of 0.78 which is substantial/positive evidence for the null model.

### Addition

The ANOVA revealed a main effect of *Time, F*(1,97) = 29.748, *p* < 0.001, η*^2^* = 0.227. Additionally, the covariate *CFT-matrices* was also significant, *F*(1,97) = 28.983, *p* < 0.001, η*^2^* = 0.227. No other main effects or interactions were significant. Comparing the null model (excluding *CFT-matrices* and *Sex* and including the main factors *Time* and *Group* as nuisance variables) with the alternative model (including the interaction of *Time* and *Group)* revealed a BF_01_ of 1.52 for the interaction and a probability of *p*(H_0_| D) = 0.60 which is weak/anecdotal evidence for the null model. Thus, for addition there is no strong evidence either for the null model or for the alternative model.

### Subtraction

Due to the fact that some children had profound difficulties in subtraction (some children were unable to solve even a single subtraction task), we excluded from analysis children who scored zero in pre- or post-tests. This reduced the original sample to 63 children (*N* = 16 in the experimental group, 5 female; *N* = 47 in the control group, 20 female). With this reduced sample, the ANOVA revealed a main effect of *Time, F*(1,58) = 13.137, *p* < 0.001, η*^2^* = 0.181. Additionally, we found an effect of *CFT-matrices*, *F*(1,58) = 25.373, *p* < 0.001, η*^2^* = 0.290. No other main effects or interactions were significant. Comparing the null model (excluding *CFT-matrices* and *Sex* and including the main factors *Time* and *Group* as nuisance variables) with the alternative model (including the interaction of *Time* and *Group)* revealed a BF_01_ of 1.64 for the interaction and a probability of *p*(H_0_| D) = 0.62 which is weak/anecdotal evidence for the null model. Thus, similarly to addition, for subtraction there is no strong evidence either for the null model or for the alternative model.

### Correlations

To characterize the relation between finger gnosis and arithmetic measures in more detail we calculated correlations and partial correlations (controlling for *CFT-matrices* scores) between all dependent measures pre- and post-test (see Supplementary Tables S4, S5). First, nearly all of our measures showed significant positive correlations pre- and post-test, respectively, as well as between pre- and post-test. This was supported by the Bayes-Factors indicating strong support for nearly all correlations compared to the null hypothesis (no correlation). However, correlations between arithmetic tasks (addition, subtraction and completion to 5/10) were consistently higher (0.61–0.79) than correlations between finger gnosis and arithmetic tasks (0.28–0.48) at pre- or post-test, respectively (see Supplementary Table S4).

### Multiple Stepwise Regression

To examine whether finger gnosis at pre-test uniquely predicts any of the arithmetic skills at post-test beyond those at pre-test we ran a multiple stepwise regression. All predictors were taken from the pre-test. For addition at the post-test the final model included two predictors: addition and subtraction, *R*^2^ = 0.66, *F*(2,101) = 93.83, *p* < 0.001. For subtraction the final model included three predictors: addition, subtraction and CFT, *R*^2^ = 0.54, *F*(3,101) = 38.39, *p* < 0.001. For completion to 5/10 the final model included three predictors: addition, subtraction and completion to 5/10, *R*^2^ = 0.46, *F*(3,101) = 28.27, *p* < 0.001. In sum, finger gnosis at pre-test did not significantly predict any dependent arithmetic measure at post-test, when other variables were included (see Supplementary Table S6 for *Beta*- and *p*-values and Supplementary Table S7 for Bayesian regression results). However, finger gnosis at pre-test did predict finger gnosis at post-test together with completion to 5/10, *R*^2^ = 0.25, *F*(2,101) = 16.74, *p* < 0.001. Despite significance, the explained variance of the finger gnosis performance at post-test was lower than that of the other dependent measures at post-test.

## Discussion

This study sought to investigate whether combined finger-number training improves early arithmetic skills, even before formal arithmetic instruction has started. To this end, we provided training to 102 children in their final year of kindergarten. The training took place every day, for 10 min, for 10 weeks. An active control group of children received phonological training for identical duration and intensity. The results indicated that all children improved in their finger gnosis and arithmetic performance from pre- to post-test. However, this was independent of the training they received.

This outcome is surprising as Frey et al. (unpublished) showed robust effects of a similar finger-based training in first graders on tasks of addition and subtraction compared to an active control group. We discuss two possible groups of arguments for these findings; the first group referring to the possible inefficiency of the numerical intervention training, and the second referring to the possible efficiency of the non-numerical active control training. Specifically, first, we discuss arguments why the training may not have been successful for this particular age group with this particular training setting and for these particular evaluation tasks. Second, we discuss arguments why the control training contained elements (like implicitly training sequences) that might have been beneficial for elementary numerical and arithmetic tasks as well. Finally, we discuss the underlying reasoning of some of our intervention choices and how they affect the results and interpretation.

### Reasons Why the Training Might Be Less Successful Than Other Finger-Number Trainings

Two of the dependent variables trained by Frey et al. (unpublished) were also directly trained in the present study: While finger gnosis training games differed from finger gnosis test items, the completion to 5/10 task was highly similar for training and tests. Nonetheless, Bayesian-Factor analysis revealed that the null model incorporating only the main effects seemed more probable compared to a model including the interaction of training group and time for both – finger gnosis and completion to 5/10 – measures. Unfortunately, Frey et al. (unpublished) tested neither finger gnosis nor completion to 5/10 at post-test, thus we cannot compare our outcomes in these measures with their training study in first graders. In contrast to our study, in the study by Gracia-Bafalluy and Noël (2008) only children in the ‘pure’ finger gnosis intervention group improved about 3.2 points in finger gnosis, but not children of the control intervention group. However, note that this effect could be due to a regression to the mean (Fischer, 2010) and might not be representative. Similarly, Jay and Betenson (2017) found a (small, but significant) increase of 1.9 points in finger gnosis only in groups receiving finger gnosis training. However, this rather small improvement might have been due to the combined group, because the authors analyzed both groups receiving finger gnosis training – single and combined group – together. It would be interesting to know whether the finger gnosis group and the combined group differed in their finger gnosis improvement. Note that in their study ‘finger gnosis training’ refers to activities that linked cardinal and ordinal properties of numbers to the fingers, i.e., they trained competencies like finger counting, finger-to-numbers relations or calculations with fingers. Thus, their training was comparable to ours. Yet, we found a similar improvement of 1.4 points for all groups, independently of the specific training. In addition, compared to the above-mentioned studies our children were on average 1 year younger – therefore, differences in training effects between those studies might also be attributable to the age and experience of the children.

Concerning the arithmetic measures, Jay and Betenson (2017) found that children receiving the combined training of finger gnosis and number games activities showed the largest gain in quantitative scores. While children from the other groups also improved in quantitative scores, their improvement was only half of that of the combined training group. Their quantitative score combined different measures. Some of these measures might be more related to the finger gnosis training (e.g., counting, adding dots on dice, splitting and combination of symbolic numbers); whereas others might be more related to the number training (e.g., ordering numbers, completion of number sequences, splitting and combination of non-symbolic numbers). It would have been interesting to see whether the finger gnosis group and the number group scored differently on subtasks combined in the quantitative score or whether children improved equally in all kinds of tasks from pre- to post-test. Indeed, the combined score might have obscured differential influences of finger gnosis (and number training) on different numerical skills. In contrast to the combined quantitative score of Gracia-Bafalluy and Noël (2008) and Jay and Betenson (2017) measured single numerical skills and children of the finger gnosis training improved in ‘draw a hand’ as well as in counting fingers, especially when larger number of fingers were involved (yet, improvement was only observed in response times, not in overall score). Finally, children improved in subitizing and ordinality score (comparing Arabic digits), but not in counting, magnitude comparison, enumeration and calculation. Thus, it might be that the influence of finger gnosis on numerical abilities comprises by far not all, but rather specific numerical skills.

Another important difference between the studies relates to the games that were trained. Moreover, these differences in training are related to the different levels of skills existing in the different age groups (kindergarteners versus primary school children). First, in the present study *addition and subtraction were not directly trained and combined with finger use* as in the study with the first graders (Frey et al., unpublished). The fact that direct training of tasks was successful in the study by Frey and colleagues is indirectly supported by the result of the number line accuracy task. Trained children showed no improvement in number line accuracy (Frey et al., unpublished). The authors argue this might be because the task is difficult to solve with the help of the fingers. Alternatively, this result could have emerged because number line accuracy was not practiced in the training; whereas addition and subtraction were directly trained. Now, turning to the *level of training*, most of the games trained in Frey’s study on first graders covered number relations; whereas our training for kindergarten children included more games tapping into ordinality and cardinality. The different focuses of the trainings were also due to the fact that kindergarten children have a less stable quantity-number concept than first graders. Thus, the kindergarteners required and received more games involving the learning and understanding of the finger-number relations and Arabic numbers; whereas the first graders received more exercises in using their fingers directly in addition and subtraction tasks. Thus, kindergarteners received only a few tasks which directly trained actual arithmetic skills, such as the tasks completion to 5/10 or double numbers. Moreover, none of the tasks in our study explicitly trained addition or subtraction. In contrast, the first graders in the study by Frey et al. (unpublished) received instruction to use their fingers directly in various addition and subtraction games. Thus, we might have missed training the critical level or modules (e.g., finger use in arithmetic tasks) as intensively as in the case of the first graders in the study by Frey et al. (unpublished). However, as kindergarteners do not have the same numerical and arithmetic requirements as first graders, we deliberately concentrated more on *preceding* stages of finger-numerical development (e.g., finger counting, finger-number mapping). This concentration on early stages of finger-number development might have had less of an effect on actual arithmetic skills.

However, we made clear that the focus of our study was to see whether finger-number precursor training in kindergarten has positive effects on arithmetic skills (in a similar way, this has claimed for phonological awareness and later reading performance). The present study establishes that was not the case. We believe that this is important, because embodied training of numbers and in particular finger training has been advocated by ourselves and others (e.g., Moeller et al., 2012) as a means to improve early mathematic skills. This does not of course, either preclude that another form of finger-number training or other forms of precursor training (e.g., board games, or embodied spatial-numerical training, cf. Fischer et al., 2011), may have lasting training effects. A crucial question for the future is which training, which training setups or maybe which combinations of numerical/arithmetic intervention in kindergarten are most successful in training numerical/arithmetic precursor abilities in children.

Note, that we trained all children to use their fingers with corresponding numbers in the same way. Children were trained to start with the thumb of their right hand and count up to the pinkie. For the numbers 6–10 the same order of the fingers of the left hand was used. One issue raised by one reviewer, was that we might have “deconstructed” finger-number associations that may have been already constructed by children. Thus, our results may be negative due to the children in the experimental group who counted using a divergent finger pattern at pre-intervention. For Western adults, Lindemann et al. (2011) observed that 87.5% started to count with the thumb up to the pinkie and used the same sequence of fingers for the other hand. Thus, the finger counting sequence seems to be similar among most people. In contrast to the finger sequence, the starting hand seems to be more equally distributed (Lindemann et al., 2011). Moreover, studies have shown that the task used to collect the finger counting routines (e.g., questionnaire versus spontaneous use) influenced the outcome (e.g., Lucidi and Thevenot, 2014). For example. Wasner et al. (2014) showed that finger counting habits can change heavily according to situated circumstances. When the typical Fischer (2008) and Lindemann et al. (2011) finger counting questionnaire was administered about 54% reported counting from left-to-right. When participants additionally had a pencil in their hand, even more, 62% reported counting from left-to-right. When now the horizontally aligned finger picture used in the Lindemann questionnaire was removed and participants had to count spontaneously, the left-to-right advantage not only disappeared but even reversed. With empty hands and no picture of hands in front of them, the majority of people (72%) started from right to left. This shows that people are not fixed in their counting habits, but very flexible. Moreover, they also change their finger to number-relationships substantially depending on whether they refer to cardinal numbers, to ordinal numbers or to a 1-1 relationship between finger and number (Wasner et al., 2015; which is the reason, why we trained all three of them). What is more, a recent study of Hohol et al. (2018) assessed the reliability and flexibility of finger counting habits. While reliability was satisfactory (about 75% reported using the same hand on both occasions), participants also reported huge flexibility. Overwhelmingly they said that they are also comfortable starting counting with the non-preferred hand, and about 50% even said that if they hold an object in their preferred starting hand, they do not bother to change hands or put the object away, but just start counting with the other hand. These studies point to a substantial flexibility in counting habits.

Nevertheless, because we tested kindergarten children, one might argue that they have less flexibility than first graders or adults tested in above studies (see Sato and Lalain, 2008; Previtali et al., 2011 for developmental data). Therefore, we reanalyzed all our data to see if there was any difference between children who were trained in congruence with their finger counting preference and those who were not. In the finger gnosis task children were asked to count to ten with their fingers. We compared two groups: one group who was trained in congruence with their preference, and the other group who was not. At pre-intervention, in the experimental group, 27 children counted in the *trained pattern* (in which 6 children switched to a divergent pattern at post-intervention), 8 children counted in a *divergent pattern* (in which 6 children switched to the trained counting pattern at post-intervention). The two groups did not differ in any of the post-tests (Mann–Whitney), *p*_(finger gnosis, completion, addition)_ > 0.65. In the reduced sample for subtraction, 11 children with (pre-intervention) *trained counting pattern* and 5 children with (pre-intervention) *divergent counting pattern* were included. They did not differ in subtraction at post-test, *p* = 0.69. Obviously, the results have to be interpreted with caution, because of the different and small sample size, but, for the moment, there was no indication that the congruency of training direction with natural habits had an effect in any analysis. These data are consistent with the flexibility shown in the studies above and clearly inconsistent with the assumption that this issue affected training success.

### Why the Null Effect Could Be Due to Improvement of the Control Intervention

One important difference between former finger training studies and our study is the *control intervention*. Frey et al. (unpublished) and Jay and Betenson (2017) had only no-intervention control groups. Gracia-Bafalluy and Noël (2008) had a story comprehension control group and a no-intervention control group. In contrast, we compared our finger-number training to a group trained in phonological awareness. Thus, domain-general factors might have improved with both kinds of trainings as well as domain-specific factors that might have overlapped in both training groups.

It is known that *domain-general variables* (e.g., concentration, attention, executive functions) can modulate performance in domain-specific skills (e.g., see Aunio and Niemivirta, 2010 how inattention modulated numerical performance). The influence of domain-general skills on specific skills might of course depend on the particular domain-general and/or domain-specific variable. For example, the causal relation between working memory and arithmetic skills is heatedly debated (Welsh et al., 2010; Melby-Lervag and Hulme, 2013; Cragg and Gilmore, 2014; Passolunghi and Costa, 2016; Honore and Noël, 2017; Ramani et al., 2017). Moreover, the strength of this relation may also depend on other factors, for example, whether children come from low-income families and/or whether children may have a risk for special impairments. Likewise, specific interventions (such as the training) provided to children in our study may have general effects on attention, concentration, motivation, working memory and other domains. Thus, what might have happened in our study is that the phonological training group was trained in general-domain variables and this, in turn, also led to improvement in their numerical skills (Purpura and Ganley, 2014; but see also Purpura and Reid, 2016).

Initially, we thought we had constructed our control trainings in such a way as there was no overlap in the training of specific skills (finger-number skills versus phonological awareness skills). However, taking a closer look at the specific exercises in both trainings may reveal certain similarities of trained *domain-specific factors*. Possible candidates are sequencing and ordinality, which both apply for numbers as well as for words (for example, one can count and order sounds in a spoken word). Thus, implicit training of these concepts in the phonological group might have generalized to the positive outcome in the numerical tasks. For example, one game in the phonological group involved counting a phoneme sequence in a word (e.g., M-U-MM-Y), which might have directly trained both ordinal-numerical as well as phoneme-skills.

This interpretation is supported by studies showing a relation between domain-general ordering skills (by using ordering of months or letters) and arithmetic skills in children (e.g., O’Connor et al., 2018) and adults (e.g., Morsanyi et al., 2017; Sasanguie et al., 2017; Vos et al., 2017).

A recent study of Xu and LeFevre (2016) show that learning sequential relations is beneficial for later arithmetic and numerical skills. It is therefore possible that more sequential finger-number games would have been beneficial for training success. Again, our non-numerical control training was also training sequential processes albeit not for numbers. As already discussed, children improved in both training conditions, the experimental and the active control training. Relating this to Xu and LeFevre (2016), one might suggest that in our control training, we have also trained sequential relations – although these relations were non-numerical, there might have been transfer effects to sequential numerical knowledge, which is an important cornerstone for later arithmetic skills. Note that in this respect our training lasted 10 weeks (Xu and LeFevre, 2016: 3 weeks), which leaves much time for implicit and explicit transfer effects.

### Finger Gnosis Was Not a Predictor in This Study

Turning to the sub-question of whether finger gnosis is a predictor for later arithmetic skills, our regression result did not support this claim. Although finger gnosis correlated with arithmetic performance, it did not uniquely predict any of our arithmetic measures. These results are in line with studies that assume that factors other than finger gnosis – namely numerical knowledge and initial arithmetic abilities – might be more important in predicting later arithmetic skills (Long et al., 2016). Still, others have shown that finger gnosis can predict at least a small variance of later arithmetic performance (Penner-Wilger et al., 2007, 2009; Kohn et al., 2015; Poltz et al., 2015; Wyschkon et al., 2015; Wasner et al., 2016). However, a combination of the young age (leading to more error variance in the testing) and other control variables may be responsible for finger gnosis not being a predictor in the current study.

### Active Control Group Rather Than Waiting List Control Group

We view as strength of our study that we used an active control group and not just a waiting control group. Note that in the child literature waiting control groups are viewed from critically to not acceptable (Fischer et al., 2013) and some authors do not include intervention studies without active control groups in their reviews (Slavin et al., 2009). The reason is that waiting control groups do not allow for the distinguishing of intervention-specific effects from intervention-unspecific effects such as attention, motivation or unspecific cognitive factors (learning how to learn) from intervention-specific effects, such as learning finger-number relations in our study. A recent meta-analysis confirmed this concern. Intervention studies without active control groups had generally larger effect sizes (Fischer et al., 2013). However, it is impossible to distinguish the contribution of intervention-unspecific and intervention-specific effects for such effect sizes. Therefore, we used an active control design and did not add a waiting control group, because it would not allow any substantial additional interpretation as regards the specific effects of our training.

### Multiple Component vs. Single-Component Interventions

When one reviews intervention studies, it is essential to distinguish between short-term interventions, where one component in one game or task is trained, and long-term interventions, where multiple components and tasks are trained (see Fischer et al., 2013, for an overview). Some of us have conducted single-component embodied interventions targeting embodied numerosity in different variations (e.g., Fischer et al., 2011, 2015; Link et al., 2013; Link et al., 2014; Dackermann et al., 2016b; for reviews see Moeller et al., 2012, 2015; Dackermann et al., 2016a, 2017). When one conducts such trainings, it is inevitable that children get bored after a very short period of time. For instance, Fischer et al. (2015) could not even include post-tests after the second training in a cross-over design, because the decreased motivation of the children caused performance to drop substantially in the second post-test.

Any long-term intervention in such young children therefore necessarily cannot rely on one component, because it would get boring for the children after a few or even one session. We are not aware of any long-term intervention in numerical cognition which lasted over 50 sessions in 10 weeks (or more) and which used only one particular game for any numerical construct. All comparable interventions we are aware of used multiple modules and multiple games to improve one or more particular conceptual representation or process. Therefore, in any (not only our) long-term intervention with kindergarten children, it will always be impossible to track down any eventual changes to one particular game or module. This is only possible in short-term interventions with very few sessions, where children do not get bored by multiple repetitions of the same simple arithmetic game.

We have included finger gnosis in our multi-component finger-number intervention program, because earlier results (e.g., Noël, 2005; Wasner et al., 2016) suggested that finger gnosis may be weakly related to arithmetic skill. However, of our whole training modules, only two short training games exclusively targeted finger gnosis, all other games were explicitly related to finger-number relations. Thus, training finger gnosis was a very small part of the multi-component intervention program and given that the relations between finger gnosis and math are weak, we do not believe that their inclusion had a large impact on the results. However, theoretically, we cannot preclude that these two of the 18 games contributed to the null effects in this study.

### Limitations

As the finger gnosis and finger-number training provided in the current study obviously was not effective beyond the control group, it might be that the training ought to be *provided together with formal arithmetic instruction*. A key difference between the kindergarten children in our study and the first graders in the study by Frey et al. (unpublished) was that the latter had already been formally introduced to the concept of addition and subtraction at school, which of course was not the case for our kindergarten children. The lack of formal arithmetic education did not prevent some of the kindergarten children from solving quite a few of the addition tasks. On the other hand, the subtraction tasks were very difficult and often frustrating for nearly all of the children. The latter was also obvious as this task showed a high fluctuation in performance. Nearly 20 percent of the children could not solve even one of the subtraction items at post-test, but the same children had solved an average of nearly five items at pre-test. This observation might be a consequence of the fact that kindergarteners are used to counting forward rather than backward. In line with this, it has been shown that preschoolers had more difficulties using a task to access the preceding compared to the next number (e.g., Sella et al., 2019; Sella and Lucangeli, 2020). In addition, the fact that basic arithmetic performance varies strongly at this age may be due to fluctuations in attention and motivation (see, e.g., Aunio and Niemivirta, 2010 for the influence of inattention on arithmetic performance). Other studies have also found large individual differences in numerical abilities in preschoolers (e.g., Weinhold Zulauf et al., 2003; Dowker, 2008).

Further, as we did not control for *external interventions* taking place at the individual kindergarten or at the homes of the children, it might be that these interventions leveled out the effects of the training. However, although often not mentioned this applies to all of the studies in the field, since no kindergarten, school and probably almost no parents would agree to participate in a longitudinal numerical study in which all numerical/arithmetic activities are forbidden for the time of the longitudinal study this might create an additional source of error variance. Additionally, stronger promotion of numerical skills in the kindergarten and/or in the family might in turn also boost numerical knowledge. This may be even reinforced by the fact that in and around the city of Tübingen, where the training took place, families have an above-average socioeconomic status, and thus, children may have been promoted even more. If many of the children in our study received a great deal of such numerical promotion in their kindergartens or families anyway in this developmental period, this could have prevented our training from having a visible additional benefit. Thus, the training may still be beneficial for (possibly lower SES) families, in which numerical skills of children are supported or promoted to a lesser extent.

It could also be the case when familiarizing kindergarteners with numbers the *increased interest* in one domain might generalize for neighboring domains like sounds and letters and vice versa, thereby promoting improvement in both fields. The finding that numerical skills obviously improve dramatically during the last kindergarten year was also shown by Weinhold Zulauf et al. (2003) who tested over 300 German-speaking children in Austria (see also Krajewski et al., 2008). The authors even speak of a *“sensitive period”* for the acquisition of numerical skills. Thus, children at this age gain knowledge in the domain of numbers very fast through natural interest.

At last, we do not want to omit the possibility that the training might have had no effect whatsoever. In this case, *overall maturation*, which is certainly fast at that age, might have led to the improvement of all skills in all groups. However, we do not think maturation plays a sole role, as other studies with waiting control groups consistently showed differences when compared to the intervention groups (e.g., Gracia-Bafalluy and Noël, 2008; Jay and Betenson, 2017; Frey et al., unpublished). Moreover, other studies focusing on other numerical precursor skills, or including a broader range of such skills, have shown intervention effects in kindergarten children (e.g., Kaufmann et al., 2005; Krajewski et al., 2008; Praet and Desoete, 2014).

## Conclusion and Perspectives

In sum, we suggest that the difference in training and age was responsible for the different outcomes between the Frey et al. (unpublished) study and the current study. The first graders in the study by Frey et al. (unpublished) had received training in number relations and direct finger use for addition and subtraction, and the kindergarten children in our study had received training in a quantity of number concepts. Both studies trained a variety of different skills occurring at different developmental stages (finger gnosis, 1-1 finger-number mapping, ordinality and cardinality of numbers and number relations in base-10 and place-value system). It may be a rather complicated but potentially rewarding task for future studies to try and disentangle these factors and test more directly which specific components of the training were responsible for the training effect in Frey et al. (unpublished) first-graders and which components might be more promising for training in kindergarteners compared to older children.

Maybe one should also take the developmental stage of the individual child into account. For example, it might be fruitful to apply an adaptive finger-based numerical training suited to the needs of the individual child (similar to, e.g., Praet and Desoete, 2014, for computerized counting), rather than having all children play the same games. Given the large individual differences in preschooler’s numerical abilities (Dowker, 2008), a lot of the games might be boring for some children but overburden others. Individual interventions carried out in primary school directly trained weak number skills of individual children (e.g., Dowker and Sigley, 2010; Holmes and Dowker, 2013). The individual arithmetical skills of the children trained in these studies were highly susceptible to the individual intervention. Some of the concepts used in the training were similar to ours (e.g., counting, written symbols, etc.), whereas others tapped more into conceptual and reasoning domains. Thus, again by comparing these interventions in primary school with our kindergarten training it is difficult to uncover the effective (or ineffective) components of our training. Differences of outcome could also be due to the different characteristics of the groups (preschool-aged normally developing children versus school-aged children with arithmetic difficulties). In sum, different outcomes could be due to the different trainings, the trained skills, or the individual adaption of the training. Finally, it could be due to a combination of all three factors. Thus, it remains for future research to find out whether, and what components of, finger-based numerical training can be trained at which ages (specifically kindergarten versus primary school) and which training might be best-suited for normally developing or at-risk children (see Kaufmann et al., 2003; Dowker and Sigley, 2010; Holmes and Dowker, 2013 for interventions in primary school children with arithmetic difficulties). Moreover, a comparison and/or combination of finger-based numerical training with other components, that have been found to be effective, e.g. conceptual training (for kindergarten children see Kaufmann et al., 2005) might be fruitful.

## Conclusion

All of our kindergarteners showed improved scores in finger-gnosis, addition, subtraction and completion to 5/10, independent of the training they received. We argue that these general improvements could have been due to both domain-general and domain-specific training effects. As our control training contained elements (such as sequencing or ordinality) that might have been beneficial for numerical skills as well a final evaluation of the training as being effective or ineffective is preliminary and may require a different active control group. Further studies investigating how finger-number trainings in kindergarten children might affect the development of numerical skills should incorporate different active intervention control groups to disentangle general and specific training effects from maturation effects and environmental factors like institutional or private promotion. Finally, as a first intervention study where finger-number associations were trained in normally developing kindergarteners, our data provide insights about the impact of finger-number associations for arithmetic development. Even though we are convinced that appropriate embodied trainings might help (e.g., Dackermann et al., 2017), it is in our view important to also publish and acknowledge the limitations of such training approaches when they were not as successful as we would have ourselves postulated before we saw the data.

## Data Availability Statement

The datasets generated for this study are available on request to the corresponding author.

## Ethics Statement

The studies involving human participants were reviewed and approved by the Ethical Committee of the German Psychological Association (“Ethikkommission der Deutschen Gesellschaft für Psychologie”, US 082014). Written informed consent to participate in this study was provided by the participants’ legal guardian/next of kin.

## Author Contributions

US conceived the study and took primary responsibility for drafting the manuscript. US, AB, and H-CN contributed to design of the training and the tasks. AB conducted the study. US analyzed the data. AB and H-CN commented on drafts. All authors contributed to the manuscript revision, read and approved the submitted version.

## Conflict of Interest

The authors declare that the research was conducted in the absence of any commercial or financial relationships that could be construed as a potential conflict of interest.

## Footnotes

1 As suggested by a reviewer we ran all ANOVA and ANCOVA analyses including Bayes for each control group, separately (see Supplementary Tables S8–S11 and Supplementary Figure S1). The results were similar to those when both control groups were merged into a single control group.

## Appendix

**TABLE A1:** Trained conceptual level, skills and games/tasks applied in the training.

Conceptual level	Skill	Game/Task	Order of occurrence in the training. Games were applied with increasing difficulty.	Occurred N-times in training
**Finger gnosis (not related to number)**	Motoric accuracy	“Finger tapping” with each hands separately and together	1	5
	Motoric divergence	“Labyrinth”: tracing a way through a labyrinth with all fingers, separately	2	4
**1-to-1 mappings of finger and number**	Verbal finger-number mapping	“Naming the fingers,” i.e., thumb, index finger, middle finger, ring finger and pinkie and mapping the right numbers (right hand - 1-5 and left hand 6-10)	3	4
	Visual finger-number mapping in association with learning visual Arabic digits	“Tracing numbers” 1-10 on a sheet with the respective finger of the right (1-thumb, 2 - index finger, 3 - middle finger, 4 - ring finger, 5 - pinkie) or left (6-thumb, 7 - index finger, 8 - middle finger, 9 - ring finger, 10 - pinkie) hand	4	3
**Ordinal finger-number associations**	Counting	“Finger counting” forwards and backwards and starting with different numbers	5	3
		“Counting objects.” Children should show the counted objects with their fingers.	12	4
		“Clapping”: counting the clapping of the trainer and other children. Children should show the number of claps with their fingers.	13	5
	Ordering (Ordinality based on cardinality)	“Train-Game” with groups of 3-5 children. Each child got an Arabic number. Children had to show their digit with their fingers and order themselves in the correct numerical sequence like train carriages without talking. The number sequences were either continuous with missing “carriages” in between, e.g., 3, 5, and 9	14	6
		“Order card desks” with fingers, digits and points into the right sequence (from 1 to 10)	15	3
**Cardinal finger-number associations**	Verbal and visual finger to number and number to finger mapping of the respective cardinalities	“Corresponding number” naming the corresponding number to shown fingers (6) The trainer showed a finger-number pattern for a few seconds while saying a rhyme. Children had to recognize the finger-number pattern and show it with their own fingers and say the corresponding number. (7) Later, single children were allowed to show a pattern and appoint another child in solving the task.	6 and 7	12
		“Story-time” detect numbers that were hidden in stories and show the cardinality of the numbers with their fingers”	8	9
		“Memory” with cards displaying fingers and numbers	9	4
		“Bingo” with finger cards Children had to mark numbers on a sheet	10	7
		“Domino” with combined finger and number cards	11	6
**Number relations: Base-10 and place-value system**	Completion to 5/10	“Completion-Game” showing how many fingers are needed to 5 and 10	16	3
		“Completion” (to 5 and to 10) with cards displaying fingers	17	4
	Double numbers	“Double numbers” children should show the double number (with the fingers) to fingers shown by the trainer	18	3
