# Supplementary material for: A Finger-Based Numerical Training Failed to Improve Arithmetic Skills in Kindergarten Children Beyond Effects of an Active Non-numerical Control Training
Source: Front Psychol. 2020 Mar 24;11:529. doi: 10.3389/fpsyg.2020.00529 (PMC7105809; doi:10.3389/fpsyg.2020.00529)
Supplement: Supplementary file 1 [file Data_Sheet_1.docx]

**Supplementary Material**

Table S1

| *Bayesian Repeated Measures ANOVA* | | | | | | | | | | | |
| --- | --- | --- | --- | --- | --- | --- | --- | --- | --- | --- | --- |
| Models | | P(M) | | P(M\|data) | | BF _M_ | | BF _01_ | | error % | |
| **FINGER GNOSIS**  Null model (incl. Finger gnosis, Group, Subject) |  | 0.500 |  | **0.835** |  | 5.063 |  | 1.000 |  |  |  |
| Finger gnosis  x  Group |  | 0.500 |  | 0.165 |  | 0.198 |  | **5.063** |  | 3.828 |  |
|  |  |  |  |  |  |  |  |  |  |  |  |
| **COMPLETION** |  |  |  |  |  |  |  |  |  |  |  |
| Null model (incl. Group, Completion, Subject) |  | 0.500 |  | **0.781** |  | 3.561 |  | 1.000 |  |  |  |
| Group x Completion |  | 0.500 |  | 0.219 |  | 0.281 |  | **3.561** |  | 2.942 |  |
|  |  |  |  |  |  |  |  |  |  |  |  |
| **ADDITION** |  |  |  |  |  |  |  |  |  |  |  |
| Null model (incl. Group, Addition, Subject) |  | 0.500 |  | **0.604** |  | 1.524 |  | 1.000 |  |  |  |
| Group x Addition |  | 0.500 |  | 0.396 |  | 0.656 |  | **1.524** |  | 4.399 |  |
|  |  |  |  |  |  |  |  |  |  |  |  |
| **SUBTRACTION** (reduced sample, N = 63) |  |  |  |  |  |  |  |  |  |  |  |
| Null model (incl. Subtraction, Group, Subject) |  | 0.500 |  | **0.621** |  | 1.636 |  | 1.000 |  |  |  |
| Subtraction x Group |  | 0.500 |  | 0.379 |  | 0.611 |  | **1.636** |  | 3.801 |  |

Table S2

| *ANCOVA* | | | | | | | | | | | | | | | | | | | | | | | | | | | | | |
| --- | --- | --- | --- | --- | --- | --- | --- | --- | --- | --- | --- | --- | --- | --- | --- | --- | --- | --- | --- | --- | --- | --- | --- | --- | --- | --- | --- | --- | --- |
| Cases | | | Sum of Squares | | | | | df | | | | | Mean Square | | | | F | | | | p | | | | | η² | | | |
| **FINGER GNOSIS_post**  Group |  | 0.314 | | |  | | 1 | | |  | | 0.314 | | |  | 0.015 | |  | 0.902 | | | |  | 0.000 | | | |  |  |
| Sex |  | 26.670 | | |  | | 1 | | |  | | 26.670 | | |  | 1.281 | |  | 0.261 | | | |  | 0.012 | | | |  |  |
| Group x Sex |  | 0.671 | | |  | | 1 | | |  | | 0.671 | | |  | 0.032 | |  | 0.858 | | | |  | 0.000 | | | |  |  |
| **Finger gnosis_pre** |  | 256.296 | | |  | | 1 | | |  | | 256.296 | | |  | **12.312** | |  | **< .001** | | | |  | **0.111** | | | |  |  |
| CFT_matrices_centered |  | 24.781 | | |  | | 1 | | |  | | 24.781 | | |  | 1.190 | |  | 0.278 | | | |  | 0.011 | | | |  |  |
| Residual |  | 1998.481 | | |  | | 96 | | |  | | 20.818 | | |  |  | |  |  | | | |  |  | | | |  |  |
|  |  |  | | |  | |  | | |  | |  | | |  |  | |  |  | | | |  |  | | | |  |  |
| **COMPLETION_post** |  |  | | |  | |  | | |  | |  | | |  |  | |  |  | | | |  |  | | | |  |  |
| Group |  | 3.975 | | |  | | 1 | | |  | | 3.975 | | |  | 0.103 | |  | 0.748 | | | |  | 0.001 | | | |  |  |
| Sex |  | 13.260 | | |  | | 1 | | |  | | 13.260 | | |  | 0.345 | |  | 0.558 | | | |  | 0.002 | | | |  |  |
| Group x Sex |  | 11.843 | | |  | | 1 | | |  | | 11.843 | | |  | 0.308 | |  | 0.580 | | | |  | 0.002 | | | |  |  |
| **CFT_matrices_centered** |  | 175.796 | | |  | | 1 | | |  | | 175.796 | | |  | **4.575** | |  | **0.035** | | | |  | **0.032** | | | |  |  |
| **Completion_pre** |  | 1518.807 | | |  | | 1 | | |  | | 1518.807 | | |  | **39.527** | |  | **< .001** | | | |  | **0.281** | | | |  |  |
| Residual |  | 3688.776 | | |  | | 96 | | |  | | 38.425 | | |  |  | |  |  | | | |  |  | | | |  |  |
|  |  |  | | |  | |  | | |  | |  | | |  |  | |  |  | | | |  |  | | | |  |  |
| **ADDITION_post** |  |  | | |  | |  | | |  | |  | | |  |  | |  |  | | | |  |  | | | |  |  |
| Group |  | 46.303 | |  | | 1 | | |  | | 46.303 | | |  | 1.536 | | | | |  | | 0.218 | | |  | | 0.007 | |  |
| Sex |  | 0.083 | |  | | 1 | | |  | | 0.083 | | |  | 0.003 | | | | |  | | 0.958 | | |  | | 0.000 | |  |
| Group x Sex |  | 46.353 | |  | | 1 | | |  | | 46.353 | | |  | 1.537 | | | | |  | | 0.218 | | |  | | 0.007 | |  |
| **Addition_pre** |  | 3795.659 | | |  | | 1 | | |  | | 3795.659 | | |  | **125.888** | |  | **< .001** | | | |  | **0.553** | | | |  |  |
| CFT_matrices_centered |  | 79.759 | | |  | | 1 | | |  | | 79.759 | | |  | 2.645 | |  | 0.107 | | | |  | 0.012 | | | |  |  |
| Residual |  | 2894.509 | | |  | | 96 | | |  | | 30.151 | | |  |  | |  |  | | | |  |  | | | |  |  |
|  |  |  | | |  | |  | | |  | |  | | |  |  | |  |  | | | |  |  | | | |  |  |
| **SUBTRACTION_post** (reduced sample, N = 63) | | | | | | | | | | | | | | | | | | | | | | | | | | | | |  |
| Group |  | 31.770 | | |  | | 1 | | |  | | 31.770 | | |  | 1.238 | |  | 0.271 | | | |  | 0.012 | | | |  |  |
| Sex |  | 12.178 | | |  | | 1 | | |  | | 12.178 | | |  | 0.475 | |  | 0.494 | | | |  | 0.005 | | | |  |  |
| Group x Sex |  | 3.635 | | |  | | 1 | | |  | | 3.635 | | |  | 0.142 | |  | 0.708 | | | |  | 0.001 | | | |  |  |
| **Subtraction_pre** |  | 974.478 | | |  | | 1 | | |  | | 974.478 | | |  | **37.974** | |  | **< .001** | | | |  | **0.383** | | | |  |  |
| CFT_matrices_centered |  | 58.189 | | |  | | 1 | | |  | | 58.189 | | |  | 2.268 | |  | 0.138 | | | |  | 0.023 | | | |  |  |
| Residual |  | 1462.720 | | |  | | 57 | | |  | | 25.662 | | |  |  | |  |  | | | |  |  | | | |  |  |

Table S3

| \| *Bayesian ANCOVA* \| \| \| \| \| \| \| \| \| \| \| \| \| --- \| --- \| --- \| --- \| --- \| --- \| --- \| --- \| --- \| --- \| --- \| --- \| \| Models \| \| P(M) \| \| P(M\|data) \| \| BF _M_ \| \| BF _01_ \| \| error % \| \| \| **FINGER GNOSIS**  Null model (incl. Finger gnosis_pre) \|  \| 0.500 \|  \| **0.820** \|  \| 4.561 \|  \| 1.000 \|  \|  \|  \| \| Group \|  \| 0.500 \|  \| 0.180 \|  \| 0.219 \|  \| **4.561** \|  \| 0.865 \|  \| \| **COMPLETION** \|  \|  \|  \|  \|  \|  \|  \|  \|  \|  \|  \| \| Null model (incl. Completion_pre) \|  \| 0.500 \|  \| **0.772** \|  \| 3.393 \|  \| 1.000 \|  \|  \|  \| \| Group \|  \| 0.500 \|  \| 0.228 \|  \| 0.295 \|  \| **3.393** \|  \| 2.632 \|  \| \| **ADDITION** \|  \|  \|  \|  \|  \|  \|  \|  \|  \|  \|  \| \| Null model (incl. Addition_pre) \|  \| 0.500 \|  \| **0.580** \|  \| 1.381 \|  \| 1.000 \|  \|  \|  \| \| Group \|  \| 0.500 \|  \| 0.420 \|  \| 0.724 \|  \| **1.381** \|  \| 1.140 \|  \| \|  \|  \|  \|  \|  \|  \|  \|  \|  \|  \|  \|  \| \| **SUBTRACTION** (reduced sample, N = 63) \| \| \| \| \| \| \| \| \| \| \|  \| \| Null model (incl. Subtraction_pre) \|  \| 0.500 \|  \| **0.658** \|  \| 1.928 \|  \| 1.000 \|  \|  \|  \| \| Group \|  \| 0.500 \|  \| 0.342 \|  \| 0.519 \|  \| **1.928** \|  \| 1.029 \|  \| |
| --- | --- | --- | --- | --- | --- | --- | --- | --- | --- | --- | --- | --- | --- | --- | --- | --- | --- | --- | --- | --- | --- | --- | --- | --- | --- | --- | --- | --- | --- | --- | --- | --- | --- | --- | --- | --- | --- | --- | --- | --- | --- | --- | --- | --- | --- | --- | --- | --- | --- | --- | --- | --- | --- | --- | --- | --- | --- | --- | --- | --- | --- | --- | --- | --- | --- | --- | --- | --- | --- | --- | --- | --- | --- | --- | --- | --- | --- | --- | --- | --- | --- | --- | --- | --- | --- | --- | --- | --- | --- | --- | --- | --- | --- | --- | --- | --- | --- | --- | --- | --- | --- | --- | --- | --- | --- | --- | --- | --- | --- | --- | --- | --- | --- | --- | --- | --- | --- | --- | --- | --- | --- | --- | --- | --- | --- | --- | --- | --- | --- | --- | --- | --- | --- | --- | --- | --- | --- | --- | --- | --- | --- | --- | --- | --- | --- | --- | --- | --- | --- | --- | --- | --- | --- | --- | --- | --- | --- | --- | --- | --- | --- | --- | --- | --- | --- | --- | --- | --- |

Table S4

*Correlations of pre- and post-tests*

| **Pearson Correlations** |  | **Pre-tests** |  |  |  | **Post-tests** |  |  |  |  |
| --- | --- | --- | --- | --- | --- | --- | --- | --- | --- | --- |
|  |  | **Fing** | **Add** | **Sub** | **Compl** | **Fing** | **Add** | **Sub** | **Compl** | **CFT_mat** |
| **Pre-tests** |  |  |  |  |  |  |  |  |  |  |
| Fing | Pearson's r | — | 0.411 | 0.281 | 0.386 | 0.367 | 0.376 | 0.306 | 0.402 | 0.294 |
|  | p-value | — | **< .001** | 0.004 | **< .001** | **< .001** | **< .001** | 0.002 | **< .001** | 0.003 |
|  | BF_10_ | — | 1095 | 7.085 | 338.2 | 146.91 | 214.2 | 15.18 | 698.3 |  |
| Add | Pearson's r |  | — | 0.641 | 0.754 | 0.437 | 0.800 | 0.658 | 0.610 | 0.459 |
|  | p-value |  | — | **< .001** | **< .001** | **< .001** | **< .001** | **< .001** | **< .001** | **< .001** |
|  | BF_10_ |  | — | 2.350e +10 | 1.010e +17 | 3957.99 | 6.953e +20 | 1.463e +11 | 9.959e  +8 |  |
| Sub | Pearson's r |  |  | — | 0.606 | 0.326 | 0.606 | 0.645 | 0.585 | 0.359 |
|  | p-value |  |  | — | **< .001** | **< .001** | **< .001** | **< .001** | **< .001** | **< .001** |
|  | BF_10_ |  |  | — | 6.957e  +8 | 30.79 | 6.918e  +8 | 3.482e +10 | 1.067e  +8 |  |
| Compl | Pearson's r |  |  |  | — | 0.459 | 0.673 | 0.561 | 0.607 | 0.386 |
|  | p-value |  |  |  | — | **< .001** | **< .001** | **< .001** | **< .001** | **< .001** |
|  | BF_10_ |  |  |  | — | 13332.62 | 8.565e +11 | 1.382e  +7 | 7.315e  +8 |  |
| **Post-tests** |  |  |  |  |  |  |  |  |  |  |
| Fing | Pearson's r |  |  |  |  | — | 0.478 | 0.380 | 0.482 | 0.206 |
|  | p-value |  |  |  |  | — | **< .001** | **< .001** | **< .001** | 0.038 |
|  | BF_10_ |  |  |  |  | — | 41438.1 | 256.02 | 52384.0 |  |
| Add | Pearson's r |  |  |  |  |  | — | 0.737 | 0.788 | 0.466 |
|  | p-value |  |  |  |  |  | — | **< .001** | **< .001** | **< .001** |
|  | BF_10_ |  |  |  |  |  | — | 6.190e +15 | 6.155e +19 |  |
| Sub | Pearson's r |  |  |  |  |  |  | — | 0.688 | 0.461 |
|  | p-value |  |  |  |  |  |  | — | **< .001** | **< .001** |
|  | BF_10_ |  |  |  |  |  |  | — | 5.942e +12 |  |
| Compl | Pearson's r |  |  |  |  |  |  |  | — | 0.398 |
|  | p-value |  |  |  |  |  |  |  | — | **< .001** |

Fing - Finger gnosis, Add - Addition, Sub - Subtraction, Compl - Completion to 5/10, CFT-mat - subtest matrices of CFT. Correlations that are significant after Bonferroni-Holm correction for multiple testing (alpha = .05/36 is .0011) are indicated in boldface. The BayesFaktor (BF_10_) indicates how many times more the given correlation predicts the data compared to the null hypothesis (no correlation).

Table S5

| *Partial correlations of pre- and post-tests* | | | | | | | | | |
| --- | --- | --- | --- | --- | --- | --- | --- | --- | --- |
| **Pearson Partial Correlations controlling for *CFT-matrices*** | | **Pre-tests** | | | | **Post-tests** | | | |
|  | | **Fing** | **Add** | **Sub** | **Compl** | **Fing** | **Add** | **Sub** | **Compl** |
| **Pre-tests** | | | | | | | | | |
| Fing | Pearson's r | — | **0.326** | .197 | **.309** | **.327** | 0.282 | 0.201 | **.325** |
|  | p-value | — | **< .001** | .048 | **= .0016** | **< .001** | = .004 | = .044 | **< .001** |
| Add | Pearson's r |  | **—** | **.575** | **.704** | **.393** | **.746** | **.566** | **.524** |
|  | p-value |  | **—** | **< .001** | **< .001** | **< .001** | **< .001** | **< .001** | **< .001** |
| Sub | Pearson's r |  |  | **—** | **.543** | .276 | **.531** | **.579** | **.517** |
|  | p-value |  |  | **—** | **< .001** | = 0.005 | **< .001** | **< .001** | **< .001** |
| Compl | Pearson's r |  |  |  | **—** | **.420** | **.604** | **.468** | **.535** |
|  | p-value |  |  |  | **—** | **< .001** | **< .001** | **< .001** | **< .001** |
| **Post-tests** | | | | | | | | | |
| Fing | Pearson's r |  |  |  |  | — | **.441** | **.328** | **.446** |
|  | p-value |  |  |  |  | — | **< .001** | **< .001** | **< .001** |
| Add | Pearson's r |  |  |  |  |  | **—** | **.665** | **.743** |
|  | p-value |  |  |  |  |  | **—** | **< .001** | **< .001** |
| Sub | Pearson's r |  |  |  |  |  |  | **—** | **.620** |
|  | p-value |  |  |  |  |  |  | **—** | **< .001** |
| Compl | Pearson's r |  |  |  |  |  |  |  | **—** |
|  | p-value |  |  |  |  |  |  |  | **—** |

Fing - Finger gnosis, Add - Addition, Sub - Subtraction, Compl - Completion to 5/10. Correlations that are significant after Bonferroni-Holm correction for multiple testing (alpha = .05/28 is .0018) are indicated in boldface.

Table S6

*Stepwise linear regression*

| Final Model |  | *B* | | *b* | | *t* | | *p* | | 2.5% | 97.5% |
| --- | --- | --- | --- | --- | --- | --- | --- | --- | --- | --- | --- |
| **FINGER GNOSIS_post** |  |  |  | |  | |  | |  | | |
| Intercept |  | 15.470 | |  | | 6.717 | | **< .001** | | 10.900 | 20.040 |
| Completion_pre |  | 0.415 | | 0.373 | | 3.958 | | **< .001** | | 0.207 | 0.623 |
| Finger gnosis_ pre |  | 0.251 | | 0.223 | | 2.365 | | **0.020** | | 0.040 | 0.461 |
|  |  |  | |  | |  | |  | |  |  |
| **COMPLETION_post** |  |  | |  | |  | |  | |  |  |
| Intercept |  | 3.723 | |  | | 3.026 | | **0.003** | | 1.282 | 6.165 |
| Addition_pre |  | 0.226 | | 0.236 | | 1.958 | | **0.053** | | -0.003 | 0.456 |
| Subtraction_pre |  | 0.309 | | 0.276 | | 2.773 | | **0.007** | | 0.088 | 0.530 |
| Completion_pre |  | 0.472 | | 0.262 | | 2.255 | | **0.026** | | 0.057 | 0.887 |
|  |  |  | |  | |  | |  | |  |  |
| **ADDITION_post** |  |  | |  | |  | |  | |  |  |
| Intercept |  | 4.776 | |  | | 4.630 | | **< .001** | | 2.730 | 6.823 |
| Addition_pre |  | 0.792 | | 0.699 | | 9.076 | | **< .001** | | 0.619 | 0.965 |
| Subtraction_pre |  | 0.209 | | 0.158 | | 2.054 | | **0.043** | | 0.007 | 0.411 |
|  |  |  | |  | |  | |  | |  |  |
| **SUBTRACTION_post** |  |  | |  | |  | |  | |  |  |
| Intercept |  | 2.885 | |  | | 2.423 | | **0.017** | | 0.522 | 5.248 |
| Addition_pre |  | 0.360 | | 0.348 | | 3.694 | | **< .001** | | 0.166 | 0.553 |
| Subtraction_pre |  | 0.434 | | 0.360 | | 4.017 | | **< .001** | | 0.220 | 0.648 |
| CFT_matrices_centered |  | 0.410 | | 0.172 | | 2.215 | | **0.029** | | 0.043 | 0.778 |

Table S7

| *Stepwise Bayesian linear regression* | | | | | | | |
| --- | --- | --- | --- | --- | --- | --- | --- |
| Effects | | P(incl) | | P(incl\|data) | | BF _Inclusion_ | |
| **FINGER GNOSIS_post** | |  | |  | |  | |
| **Finger gnosis_pre** |  | **0.500** |  | **0.705** |  | 2.388 |  |
| Addition_pre |  | 0.500 |  | 0.442 |  | 0.794 |  |
| Subtraction_pre |  | 0.500 |  | 0.222 |  | 0.286 |  |
| **Completion_pre** |  | **0.500** |  | **0.822** |  | 4.610 |  |
| CFT_matrices_centered |  | 0.500 |  | 0.202 |  | 0.253 |  |
|  |  |  |  |  |  |  |  |
| **COMPLETION_post** |  |  |  |  |  |  |  |
| Finger gnosis_pre |  | 0.500 |  | 0.563 |  | 1.286 |  |
| Addition_pre |  | 0.500 |  | 0.545 |  | 1.198 |  |
| **Subtraction_pre** |  | **0.500** |  | **0.934** |  | **14.219** |  |
| **Completion_pre** |  | **0.500** |  | **0.789** |  | **3.747** |  |
| CFT_matrices_centered |  | 0.500 |  | 0.341 |  | 0.517 |  |
|  |  |  |  |  |  |  |  |
| **ADDITION_post** |  |  |  |  |  |  |  |
| Finger gnosis_pre |  | 0.500 |  | 0.127 |  | 0.145 |  |
| **Addition_pre** |  | **0.500** |  | **1.000** |  | **9.104e +6** |  |
| Subtraction_pre |  | 0.500 |  | 0.382 |  | 0.618 |  |
| Completion_pre |  | 0.500 |  | 0.271 |  | 0.372 |  |
| CFT_matrices_centered |  | 0.500 |  | 0.328 |  | 0.487 |  |
|  |  |  |  |  |  |  |  |
| **SUBTRACTION_post** |  |  |  |  |  |  |  |
| Finger gnosis_pre |  | 0.500 |  | 0.148 |  | 0.173 |  |
| **Addition_pre** |  | **0.500** |  | **0.978** |  | **43.646** |  |
| **Subtraction_pre** |  | **0.500** |  | **0.995** |  | **216.915** |  |
| Completion_pre |  | 0.500 |  | 0.160 |  | 0.191 |  |
| **CFT_matrices_centered** |  | **0.500** |  | **0.609** |  | **1.559** |  |
|  |  |  |  |  |  |  |  |

Table S8

**Experimental group tested against first (N37) and second (N30) control group (CG), separately**

Independent *t*-tests showed that there was no hint of pre-test differences between experimental and first (*t*_all_ ≤ 1.202, *p* ≥ .277) and second control group (*t*_all_ ≤ 0.899, *p* ≥ .372) for all tasks. Differences to the analysis with the whole control group are marked in grey.

| *Repeated Measures ANOVA* | | | | | | | | | | | | | | | | | | | | | | |  |  |  |  |  |  |  |  |  |  |  |  |  |
| --- | --- | --- | --- | --- | --- | --- | --- | --- | --- | --- | --- | --- | --- | --- | --- | --- | --- | --- | --- | --- | --- | --- | --- | --- | --- | --- | --- | --- | --- | --- | --- | --- | --- | --- | --- |
| Cases | | Sum of Squares | | | | | df | | | Mean Square | | | | F | | | p | | | | η² | |  |  |  |  |  |  |  |  |  |  |  |  |  |
| **FINGER GNOSIS**  **FIRST CG** |  | 64.101 | |  | | 1 | | |  | 64.101 | |  | **5.055** | |  | **0.028** | | |  | **0.066** | | |  |  |  |  |  |  |  |  |  |  |  |  |  |
| Finger gnosis ✻ Group |  | 0.148 | |  | | 1 | | |  | 0.148 | |  | 0.012 | |  | 0.914 | | |  | 0.000 | | |  |  |  |  |  |  |  |  |  |  |  |  |  |
| Finger gnosis ✻ Sex |  | 28.500 | |  | | 1 | | |  | 28.500 | |  | 2.247 | |  | 0.139 | | |  | 0.030 | | |  |  |  |  |  |  |  |  |  |  |  |  |  |
| Finger gnosis ✻ CFT_matrices_centered |  | 23.148 | |  | | 1 | | |  | 23.148 | |  | 1.825 | |  | 0.181 | | |  | 0.024 | | |  |  |  |  |  |  |  |  |  |  |  |  |  |
| Finger gnosis ✻ Group ✻ Sex |  | 0.298 | |  | | 1 | | |  | 0.298 | |  | 0.023 | |  | 0.879 | | |  | 0.000 | | |  |  |  |  |  |  |  |  |  |  |  |  |  |
| Residual |  | 849.617 | |  | | 67 | | |  | 12.681 | |  |  | |  |  | | |  |  | | |  |  |  |  |  |  |  |  |  |  |  |  |  |
| Group |  | 1.404 | |  | | 1 | | |  | 1.404 | |  | 0.050 | |  | 0.824 | | |  | 0.001 | | |  |  |  |  |  |  |  |  |  |  |  |  |  |
| Sex |  | 0.207 | |  | | 1 | | |  | 0.207 | |  | 0.007 | |  | 0.932 | | |  | 0.000 | | |  |  |  |  |  |  |  |  |  |  |  |  |  |
| CFT_matrices_centered |  | 86.576 | |  | | 1 | | |  | 86.576 | |  | **3.079** | |  | **0.084** | | |  | **0.044** | | |  |  |  |  |  |  |  |  |  |  |  |  |  |
| Group ✻ Sex |  | 2.767 | |  | | 1 | | |  | 2.767 | |  | 0.098 | |  | 0.755 | | |  | 0.001 | | |  |  |  |  |  |  |  |  |  |  |  |  |  |
| Residual |  | 1883.716 | |  | | 67 | | |  | 28.115 | |  |  | |  |  | | |  |  | | |  |  |  |  |  |  |  |  |  |  |  |  |  |
| **SECOND CG**  Finger gnosis |  | 48.720 | |  | | 1 | | |  | 48.720 | |  | **3.275** | |  | **0.075** | | |  | **0.051** | | |  |  |  |  |  |  |  |  |  |  |  |  |  |
| Finger gnosis ✻ Group |  | 0.935 | |  | | 1 | | |  | 0.935 | |  | 0.063 | |  | 0.803 | | |  | 0.001 | | |  |  |  |  |  |  |  |  |  |  |  |  |  |
| Finger gnosis ✻ Sex |  | 9.723 | |  | | 1 | | |  | 9.723 | |  | 0.654 | |  | 0.422 | | |  | 0.010 | | |  |  |  |  |  |  |  |  |  |  |  |  |  |
| Finger gnosis ✻ CFT_matrices_centered |  | 1.107 | |  | | 1 | | |  | 1.107 | |  | 0.074 | |  | 0.786 | | |  | 0.001 | | |  |  |  |  |  |  |  |  |  |  |  |  |  |
| Finger gnosis ✻ Group ✻ Sex |  | 2.228 | |  | | 1 | | |  | 2.228 | |  | 0.150 | |  | 0.700 | | |  | 0.002 | | |  |  |  |  |  |  |  |  |  |  |  |  |  |
| Residual |  | 892.529 |  | | 60 | | |  | 14.875 | |  |  | | | | |  |  | |  | |  |  | | | |  |  |  |  |  |  |  |  |  |
| Group |  | 2.107 |  | | 1 | | |  | 2.107 | |  | 0.065 | | | | |  | 0.799 | |  | | 0.001 |  |  |  |  |  |  |  |  |  |  |  |  |  |
| Sex |  | 0.003 |  | | 1 | | |  | 0.003 | |  | 8.890e -5 | | | | |  | 0.993 | |  | | 0.000 |  | | | |  |  |  |  |  |  |  |  |  |
| CFT_matrices_centered |  | 189.381 |  | | 1 | | |  | 189.381 | |  | **5.875** | | | | |  | **0.018** | |  | | **0.089** |  | | | |  |  |  |  |  |  |  |  |  |
| Group ✻ Sex |  | 1.509 | |  | | 1 | | |  | 1.509 | |  | 0.047 | |  | 0.829 | | |  | 0.001 | | |  |  |  |  |  |  |  |  |  |  |  |  |  |
| Residual |  | 1934.007 | |  | | 60 | | |  | 32.233 | |  |  | |  |  | | |  |  | | |  |  |  |  |  |  |  |  |  |  |  |  |  |
| **---------------------------------------------------------------------------------------------------------------** | | | | | | | | | | | | | | | | | | | | | | |  |  |  |  |  |  |  |  |  |  |  |  |  |
| **COMPLETION**  **FIRST CG** |  | 661.286 | |  | | 1 | | |  | 661.286 | |  | **32.397** | |  | **< .001** | | |  | **0.313** | | |  |  |  |  |  |  |  |  |  |  |  |  |  |
| Completion ✻ Group |  | 0.468 | |  | | 1 | | |  | 0.468 | |  | 0.023 | |  | 0.880 | | |  | 0.000 | | |  |  |  |  |  |  |  |  |  |  |  |  |  |
| Completion ✻ Sex |  | 8.299 | |  | | 1 | | |  | 8.299 | |  | 0.407 | |  | 0.526 | | |  | 0.004 | | |  |  |  |  |  |  |  |  |  |  |  |  |  |
| Completion ✻ CFT_matrices_centered |  | 64.672 | |  | | 1 | | |  | 64.672 | |  | **3.168** | |  | **0.080** | | |  | **0.031** | | |  |  |  |  |  |  |  |  |  |  |  |  |  |
| Completion ✻ Group ✻ Sex |  | 8.154 | |  | | 1 | | |  | 8.154 | |  | 0.399 | |  | 0.530 | | |  | 0.004 | | |  |  |  |  |  |  |  |  |  |  |  |  |  |
| Residual |  | 1367.619 | |  | | 67 | | |  | 20.412 | |  |  | |  |  | | |  |  | | |  |  |  |  |  |  |  |  |  |  |  |  |  |
| Group |  | 0.226 | |  | | 1 | | |  | 0.226 | |  | 0.005 | |  | 0.945 | | |  | 0.000 | | |  |  |  |  |  |  |  |  |  |  |  |  |  |
| Sex |  | 2.021 | |  | | 1 | | |  | 2.021 | |  | 0.043 | |  | 0.836 | | |  | 0.001 | | |  |  |  |  |  |  |  |  |  |  |  |  |  |
| CFT_matrices_centered |  | 755.500 | |  | | 1 | | |  | 755.500 | |  | **16.189** | |  | **< .001** | | |  | **0.192** | | |  |  |  |  |  |  |  |  |  |  |  |  |  |
| Group ✻ Sex |  | 47.832 | |  | | 1 | | |  | 47.832 | |  | 1.025 | |  | 0.315 | | |  | 0.012 | | |  |  |  |  |  |  |  |  |  |  |  |  |  |
| Residual |  | 3126.794 | |  | | 67 | | |  | 46.669 | |  |  | |  |  | | |  |  | | |  |  |  |  |  |  |  |  |  |  |  |  |  |
| **SECOND CG**  Completion |  | 674.832 | |  | | 1 | | |  | 674.832 | |  | **33.341** | |  | **< .001** | | |  | **0.342** | | |  |  |  |  |  |  |  |  |  |  |  |  |  |
| Completion ✻ Group |  | 3.151 | |  | | 1 | | |  | 3.151 | |  | 0.156 | |  | 0.695 | | |  | 0.002 | | |  |  |  |  |  |  |  |  |  |  |  |  |  |
| Completion ✻ Sex |  | 1.991 | |  | | 1 | | |  | 1.991 | |  | 0.098 | |  | 0.755 | | |  | 0.001 | | |  |  |  |  |  |  |  |  |  |  |  |  |  |
| Completion ✻ CFT_matrices_centered |  | 75.081 | |  | | 1 | | |  | 75.081 | |  | **3.710** | |  | **0.059** | | |  | **0.038** | | |  |  |  |  |  |  |  |  |  |  |  |  |  |
| Completion ✻ Group ✻ Sex |  | 1.911 | |  | | 1 | | |  | 1.911 | |  | 0.094 | |  | 0.760 | | |  | 0.001 | | |  |  |  |  |  |  |  |  |  |  |  |  |  |
| Residual |  | 1214.416 | |  | | 60 | | |  | 20.240 | |  |  | |  |  | | |  |  | | |  |  |  |  |  |  |  |  |  |  |  |  |  |
| Group |  | 50.05 | |  | | 1 | | |  | 50.05 | |  | 0.917 | |  | 0.342 | | |  | 0.012 | | |  |  |  |  |  |  |  |  |  |  |  |  |  |
| Sex |  | 20.90 | |  | | 1 | | |  | 20.90 | |  | 0.383 | |  | 0.538 | | |  | 0.005 | | |  |  |  |  |  |  |  |  |  |  |  |  |  |
| CFT_matrices_centered |  | 860.02 | |  | | 1 | | |  | 860.02 | |  | **15.753** | |  | **< .001** | | |  | **0.204** | | |  |  |  |  |  |  |  |  |  |  |  |  |  |
| Group ✻ Sex |  | 11.80 | |  | | 1 | | |  | 11.80 | |  | 0.216 | |  | 0.644 | | |  | 0.003 | | |  |  |  |  |  |  |  |  |  |  |  |  |  |
| Residual |  | 3275.57 | |  | | 60 | | |  | 54.59 | |  |  | |  |  | | |  |  | | |  |  |  |  |  |  |  |  |  |  |  |  |  |
| **---------------------------------------------------------------------------------------------------------------** | | | | | | | | | | | | | | | | | | | | | | |  |  |  |  |  |  |  |  |  |  |  |  |  |
| **ADDITION**  **FIRST CG** |  | 296.078 | |  | | 1 | | |  | 296.078 | |  | **16.807** | |  | **< .001** | | |  | **0.197** | | |  |  |  |  |  |  |  |  |  |  |  |  |  |
| Addition ✻ Group |  | 7.976 | |  | | 1 | | |  | 7.976 | |  | 0.453 | |  | 0.503 | | |  | 0.005 | | |  |  |  |  |  |  |  |  |  |  |  |  |  |
| Addition ✻ Sex |  | 1.817 | |  | | 1 | | |  | 1.817 | |  | 0.103 | |  | 0.749 | | |  | 0.001 | | |  |  |  |  |  |  |  |  |  |  |  |  |  |
| Addition ✻ CFT_matrices_centered |  | 0.426 | |  | | 1 | | |  | 0.426 | |  | 0.024 | |  | 0.877 | | |  | 0.000 | | |  |  |  |  |  |  |  |  |  |  |  |  |  |
| Addition ✻ Group ✻ Sex |  | 15.168 | |  | | 1 | | |  | 15.168 | |  | 0.861 | |  | 0.357 | | |  | 0.010 | | |  |  |  |  |  |  |  |  |  |  |  |  |  |
| Residual |  | 1180.314 | |  | | 67 | | |  | 17.617 | |  |  | |  |  | | |  |  | | |  |  |  |  |  |  |  |  |  |  |  |  |  |
| Group |  | 172.19 | |  | | 1 | | |  | 172.19 | |  | 1.714 | |  | 0.195 | | |  | 0.019 | | |  |  |  |  |  |  |  |  |  |  |  |  |  |
| Sex |  | 15.14 | |  | | 1 | | |  | 15.14 | |  | 0.151 | |  | 0.699 | | |  | 0.002 | | |  |  |  |  |  |  |  |  |  |  |  |  |  |
| CFT_matrices_centered |  | 2126.33 | |  | | 1 | | |  | 2126.33 | |  | **21.172** | |  | **< .001** | | |  | **0.230** | | |  |  |  |  |  |  |  |  |  |  |  |  |  |
| Group ✻ Sex |  | 217.12 | |  | | 1 | | |  | 217.12 | |  | 2.162 | |  | 0.146 | | |  | 0.023 | | |  |  |  |  |  |  |  |  |  |  |  |  |  |
| Residual |  | 6729.05 | |  | | 67 | | |  | 100.43 | |  |  | |  |  | | |  |  | | |  |  |  |  |  |  |  |  |  |  |  |  |  |
| **SECOND CG**  Addition |  | 396.676 | |  | | 1 | | |  | 396.676 | |  | **24.953** | |  | **< .001** | | |  | **0.276** | | |  |  |  |  |  |  |  |  |  |  |  |  |  |
| Addition ✻ Group |  | 32.728 | |  | | 1 | | |  | 32.728 | |  | 2.059 | |  | 0.157 | | |  | 0.023 | | |  |  |  |  |  |  |  |  |  |  |  |  |  |
| Addition ✻ Sex |  | 2.833 | |  | | 1 | | |  | 2.833 | |  | 0.178 | |  | 0.674 | | |  | 0.002 | | |  |  |  |  |  |  |  |  |  |  |  |  |  |
| Addition ✻ CFT_matrices_centered |  | 5.984 | |  | | 1 | | |  | 5.984 | |  | 0.376 | |  | 0.542 | | |  | 0.004 | | |  |  |  |  |  |  |  |  |  |  |  |  |  |
| Addition ✻ Group ✻ Sex |  | 44.961 | |  | | 1 | | |  | 44.961 | |  | 2.828 | |  | 0.098 | | |  | 0.031 | | |  |  |  |  |  |  |  |  |  |  |  |  |  |
| Group |  | 0.058 | |  | | 1 | | |  | 0.058 | |  | 4.960e -4 | |  | 0.982 | | |  | 0.000 | | |  |  |  |  |  |  |  |  |  |  |  |  |  |
| Sex |  | 50.734 | |  | | 1 | | |  | 50.734 | |  | 0.433 | |  | 0.513 | | |  | 0.006 | | |  |  |  |  |  |  |  |  |  |  |  |  |  |
| CFT_matrices_centered |  | 2085.654 | |  | | 1 | | |  | 2085.654 | |  | **17.813** | |  | **< .001** | | |  | **0.227** | | |  |  |  |  |  |  |  |  |  |  |  |  |  |
| Group ✻ Sex |  | 10.772 | |  | | 1 | | |  | 10.772 | |  | 0.092 | |  | 0.763 | | |  | 0.001 | | |  |  |  |  |  |  |  |  |  |  |  |  |  |
| Residual |  | 7025.094 | |  | | 60 | | |  | 117.085 | |  |  | |  |  | | |  |  | | |  |  |  |  |  |  |  |  |  |  |  |  |  |
| **---------------------------------------------------------------------------------------------------------------** | | | | | | | | | | | | | | | | | | | | | | |  |  |  |  | |  |  |  |  |  |  |  |  |
| **SUBTRACTION**  **FIRST CG** (reduced sample N=30) |  | 180.403 | |  | | 1 | | |  | 180.403 | |  | **11.302** | |  | **0.002** | | |  | **0.209** | | |  |  |  |  |  |  |  |  |  |  |  |  |  |
| Subtraction ✻ Group |  | 20.839 | |  | | 1 | | |  | 20.839 | |  | 1.305 | |  | 0.260 | | |  | 0.024 | | |  |  |  |  |  |  |  |  |  |  |  |  |  |
| Subtraction ✻ Sex |  | 9.115e -4 | |  | | 1 | | |  | 9.115e -4 | |  | 5.710e -5 | |  | 0.994 | | |  | 0.000 | | |  |  |  |  |  |  |  |  |  |  |  |  |  |
| Subtraction ✻ CFT_matrices_centered |  | 4.172 | |  | | 1 | | |  | 4.172 | |  | 0.261 | |  | 0.612 | | |  | 0.005 | | |  |  |  |  |  |  |  |  |  |  |  |  |  |
| Subtraction ✻ Group ✻ Sex |  | 1.286 | |  | | 1 | | |  | 1.286 | |  | 0.081 | |  | 0.778 | | |  | 0.001 | | |  |  |  |  |  |  |  |  |  |  |  |  |  |
| Residual |  | 654.468 | |  | | 41 | | |  | 15.963 | |  |  | |  |  | | |  |  | | |  |  |  |  |  |  |  |  |  |  |  |  |  |
| Group |  | 13.287 | |  | | 1 | | |  | 13.287 | |  | 0.233 | |  | 0.632 | | |  | 0.003 | | |  |  |  |  |  |  |  |  |  |  |  |  |  |
| Sex |  | 211.850 | |  | | 1 | | |  | 211.850 | |  | **3.709** | |  | **0.061** | | |  | **0.054** | | |  |  |  |  |  |  |  |  |  |  |  |  |  |
| CFT_matrices_centered |  | 1318.141 | |  | | 1 | | |  | 1318.141 | |  | **23.076** | |  | **< .001** | | |  | **0.339** | | |  |  |  |  |  |  |  |  |  |  |  |  |  |
| Group ✻ Sex |  | 4.707 | |  | | 1 | | |  | 4.707 | |  | 0.082 | |  | 0.776 | | |  | 0.001 | | |  |  |  |  |  |  |  |  |  |  |  |  |  |
| Residual |  | 2341.966 | |  | | 41 | | |  | 57.121 | |  |  | |  |  | | |  |  | | |  |  |  |  |  |  |  |  |  |  |  |  |  |
| **SECONG CG** (reduced sample N=17)  **SUBTRACTION** |  | 100.493 | |  | | 1 | | |  | 100.493 | |  | **9.330** | |  | **0.005** | | |  | **0.242** | | |  |  |  |  |  |  |  |  |  |  |  |  |  |
| Subtraction ✻ Group |  | 3.379 | |  | | 1 | | |  | 3.379 | |  | 0.314 | |  | 0.580 | | |  | 0.008 | | |  |  |  |  |  |  |  |  |  |  |  |  |  |
| Subtraction ✻ Sex |  | 2.831 | |  | | 1 | | |  | 2.831 | |  | 0.263 | |  | 0.612 | | |  | 0.007 | | |  |  |  |  |  |  |  |  |  |  |  |  |  |
| Subtraction ✻ CFT_matrices_centered |  | 0.047 | |  | | 1 | | |  | 0.047 | |  | 0.004 | |  | 0.948 | | |  | 0.000 | | |  |  |  |  |  |  |  |  |  |  |  |  |  |
| Subtraction ✻ Group ✻ Sex |  | 7.729 | |  | | 1 | | |  | 7.729 | |  | 0.718 | |  | 0.404 | | |  | 0.019 | | |  |  |  |  |  |  |  |  |  |  |  |  |  |
| Residual |  | 301.586 | |  | | 28 | | |  | 10.771 | |  |  | |  |  | | |  |  | | |  |  |  |  |  |  |  |  |  |  |  |  |  |
| Group |  | 34.75 | |  | | 1 | | |  | 34.75 | |  | 0.518 | |  | 0.478 | | |  | 0.012 | | |  |  |  |  |  |  |  |  |  |  |  |  |  |
| Sex |  | 132.45 | |  | | 1 | | |  | 132.45 | |  | 1.974 | |  | 0.171 | | |  | 0.045 | | |  |  |  |  |  |  |  |  |  |  |  |  |  |
| CFT_matrices_centered |  | 910.12 | |  | | 1 | | |  | 910.12 | |  | **13.566** | |  | **< .001** | | |  | **0.307** | | |  |  |  |  |  |  |  |  |  |  |  |  |  |
| Group ✻ Sex |  | 11.94 | |  | | 1 | | |  | 11.94 | |  | 0.178 | |  | 0.676 | | |  | 0.004 | | |  |  |  |  |  |  |  |  |  |  |  |  |  |
| Residual |  | 1878.48 | |  | | 28 | | |  | 67.09 | |  |  | |  |  | | |  |  | | |  |  |  |  |  |  |  |  |  |  |  |  |  |

####
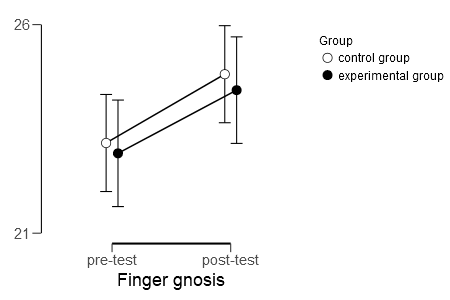

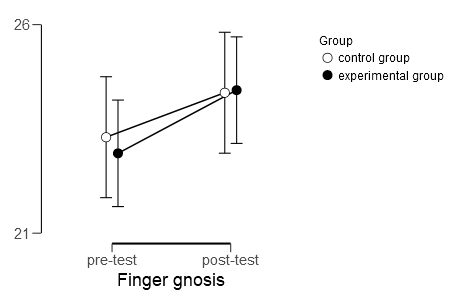

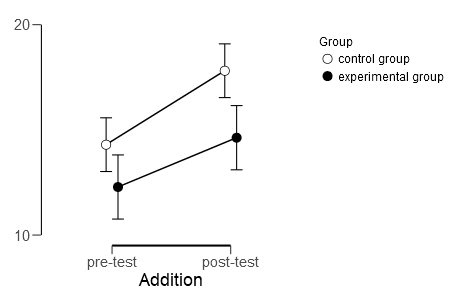

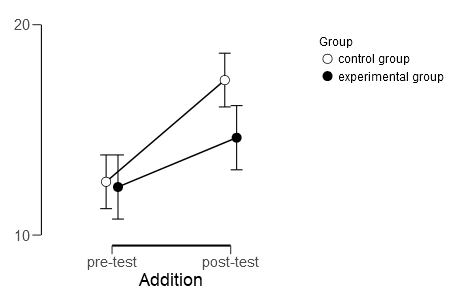

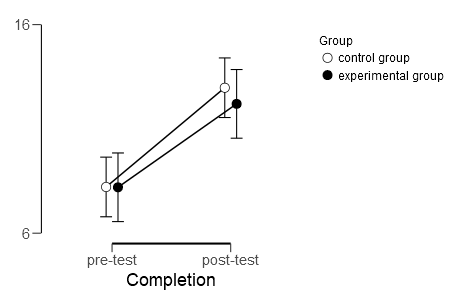

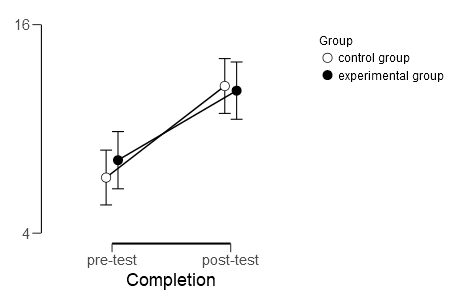

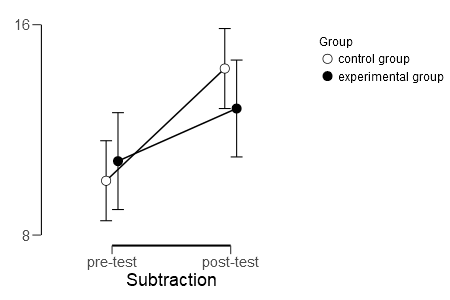

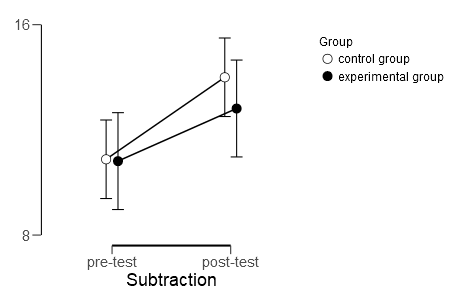


**Figure S1.** The left panel shows the comparison of experimental group with the first control group, the right panel shows the comparison with the second control group. Mean scores for all dependent variables (Finger gnosis, Completion, Addition and Subtraction) for each group (experimental group [black] versus control group [white]) and each time (pre- versus post-Test). Error bars indicate standard errors. Note, that the figure for subtraction displays results of the reduced sample (see table).

Table S9

| *Bayesian Repeated Measures ANOVA* | | | | | | | | | | |  |  |
| --- | --- | --- | --- | --- | --- | --- | --- | --- | --- | --- | --- | --- |
| Models | | P(M) | | P(M\|data) | | BF _M_ | BF _01_ | | error % | |  |  |
| **FINGER GNOSIS - FIRST CG**  Null model (incl. Finger gnosis, Group, Subject) |  | 0.500 |  | **0.803** |  | 4.074 |  | 1.000 |  |  | |  |
| Finger gnosis  ✻  Group |  | 0.500 |  | 0.197 |  | 0.245 |  | **4.074** |  | 3.909 | |  |
| **SECOND CG**  Null model (incl. Finger gnosis, Group, Subject) |  | 0.500 |  | **0.799** |  | 3.977 |  | 1.000 |  |  | |  |
| Finger gnosis  ✻  Group |  | 0.500 |  | 0.201 |  | 0.251 |  | **3.977** |  | 4.405 | |  |
| ------------------------------------------------------------------------------------------------------------------------- | | | | | | | | | | | | |
| **COMPLETION - FIRST CG**  Null model (incl. Completion, Group, subject) |  | 0.500 |  | **0.765** |  | 3.253 |  | 1.000 |  |  | |  |
| Completion  ✻  Group |  | 0.500 |  | 0.235 |  | 0.307 |  | **3.253** |  | 10.22 | |  |
| **SECOND CG**  Null model (incl. Completion, Group, subject) |  | 0.500 |  | **0.761** |  | 3.193 |  | 1.000 |  |  | |  |
| Completion  ✻  Group |  | 0.500 |  | 0.239 |  | 0.313 |  | **3.193** |  | 2.360 | |  |
| ------------------------------------------------------------------------------------------------------------------------- | | | | | | | | | | | | |
| **ADDITION - FIRST CG**  Null model (incl. Addition, Group, subject) |  | 0.500 |  | **0.786** |  | 3.676 |  | 1.000 |  |  | |  |
| Addition  ✻  Group |  | 0.500 |  | 0.214 |  | 0.272 |  | **3.676** |  | 13.87 | |  |
| **SECOND CG**  Null model (incl. Group, Addition, subject) |  | 0.500 |  | **0.507** |  | 1.028 |  | 1.000 |  |  | |  |
| Group  ✻  Addition |  | 0.500 |  | 0.493 |  | 0.973 |  | **1.028** |  | 4.116 | |  |
| ------------------------------------------------------------------------------------------------------------------------- | | | | | | | | | | | | |
| **SUBTRACTION** (reduced sample) **- FIRST CG**  Null model (incl. Subtraction, Group, subject) |  | 0.500 |  | **0.596** |  | 1.473 |  | 1.000 |  |  | |  |
| Subtraction  ✻  Group |  | 0.500 |  | 0.404 |  | 0.679 |  | **1.473** |  | 3.316 | |  |
| **SECOND CG**  Null model (incl. Group, Subtraction, subject) |  | 0.500 |  | **0.713** |  | 2.484 |  | 1.000 |  |  | |  |
| Group  ✻  Subtraction |  | 0.500 |  | 0.287 |  | 0.403 |  | **2.484** |  | 3.498 | |  |

Table S10

| *ANCOVA* | | | | | | | | | | | | | | | | | | | | | | | | | | | | | |
| --- | --- | --- | --- | --- | --- | --- | --- | --- | --- | --- | --- | --- | --- | --- | --- | --- | --- | --- | --- | --- | --- | --- | --- | --- | --- | --- | --- | --- | --- |
| Cases | | | Sum of Squares | | | | | df | | | | | Mean Square | | | | F | | | | p | | | | | η² | | | |
| **FINGER GNOSIS_post - FIRST CG**  Group |  | 0.741 | | |  | | 1 | | |  | | 0.741 | | |  | 0.037 | |  | 0.849 | | | |  | 0.000 | | | |  |  |
| Sex |  | 27.216 | | |  | | 1 | | |  | | 27.216 | | |  | 1.351 | |  | 0.249 | | | |  | 0.017 | | | |  |  |
| Group ✻ Sex |  | 0.011 | | |  | | 1 | | |  | | 0.011 | | |  | 5.354e -4 | |  | 0.982 | | | |  | 0.000 | | | |  |  |
| Finger gnosis_pre |  | 227.255 | | |  | | 1 | | |  | | 227.255 | | |  | **11.280** | |  | **0.001** | | | |  | **0.143** | | | |  |  |
| CFT_matrices_centered |  | 1.349 | | |  | | 1 | | |  | | 1.349 | | |  | 0.067 | |  | 0.797 | | | |  | 0.001 | | | |  |  |
| Residual |  | 1329.690 | | |  | | 66 | | |  | | 20.147 | | |  |  | |  |  | | | |  |  | | | |  |  |
| **SECOND CG**  Group |  | 2.408 | | |  | | 1 | | |  | | 2.408 | | |  | 0.097 | |  | 0.756 | | | |  | 0.001 | | | |  |  |
| Sex |  | 10.211 | | |  | | 1 | | |  | | 10.211 | | |  | 0.413 | |  | 0.523 | | | |  | 0.006 | | | |  |  |
| Group ✻ Sex |  | 4.041 | | |  | | 1 | | |  | | 4.041 | | |  | 0.163 | |  | 0.687 | | | |  | 0.002 | | | |  |  |
| Finger gnosis_ pre |  | 240.433 | | |  | | 1 | | |  | | 240.433 | | |  | **9.728** | |  | **0.003** | | | |  | **0.139** | | | |  |  |
| CFT_matrices_centered |  | 15.701 | | |  | | 1 | | |  | | 15.701 | | |  | 0.635 | |  | 0.429 | | | |  | 0.009 | | | |  |  |
| Residual |  | 1458.266 | | |  | | 59 | | |  | | 24.716 | | |  |  | |  |  | | | |  |  | | | |  |  |
| ----------------------------------------------------------------------------------------------------------------------- | | | | | | | | | | | | | | | | | | | | | | | | | | | |  |  |
| **COMPLETION_post - FIRST CG**  Group |  | 0.910 | | |  | | 1 | | |  | | 0.910 | | |  | 0.022 | |  | 0.882 | | | |  | 0.000 | | | |  |  |
| Sex |  | 15.819 | | |  | | 1 | | |  | | 15.819 | | |  | 0.383 | |  | 0.538 | | | |  | 0.004 | | | |  |  |
| Group ✻ Sex |  | 11.055 | | |  | | 1 | | |  | | 11.055 | | |  | 0.268 | |  | 0.607 | | | |  | 0.003 | | | |  |  |
| Completion_ pre |  | 799.074 | |  | | 1 | | |  | | 799.074 | | |  | **19.339** | | | | |  | | **< .001** | | |  | | **0.217** | |  |
| CFT_matrices_centered |  | 133.491 | |  | | 1 | | |  | | 133.491 | | |  | **3.231** | | | | |  | | **0.077** | | |  | | **0.036** | |  |
| Residual |  | 2727.127 | |  | | 66 | | |  | | 41.320 | | |  |  | | | | |  | |  | | |  | |  | |  |
| **SECOND CG**  Group |  | 6.103 | | |  | | 1 | | |  | | 6.103 | | |  | 0.148 | |  | 0.702 | | | |  | 0.002 | | | |  |  |
| Sex |  | 3.953 | | |  | | 1 | | |  | | 3.953 | | |  | 0.096 | |  | 0.758 | | | |  | 0.001 | | | |  |  |
| Group ✻ Sex |  | 3.794 | | |  | | 1 | | |  | | 3.794 | | |  | 0.092 | |  | 0.763 | | | |  | 0.001 | | | |  |  |
| Completion_ pre |  | 1031.677 | | |  | | 1 | | |  | | 1031.677 | | |  | **25.061** | |  | **< .001** | | | |  | **0.287** | | | |  |  |
| CFT_matrices_centered |  | 124.060 | | |  | | 1 | | |  | | 124.060 | | |  | **3.014** | |  | **0.088** | | | |  | **0.034** | | | |  |  |
| Residual |  | 2428.831 | | |  | | 59 | | |  | | 41.167 | | |  |  | |  |  | | | |  |  | | | |  |  |
| ----------------------------------------------------------------------------------------------------------------------- | | | | | | | | | | | | | | | | | | | | | | | | | | | |  |  |
| **ADDITION_post - FIRST CG**  Group |  | 18.784 | | |  | | 1 | | |  | | 18.784 | | |  | 0.527 | |  | 0.470 | | | |  | 0.004 | | | |  |  |
| Sex |  | 3.995 | | |  | | 1 | | |  | | 3.995 | | |  | 0.112 | |  | 0.739 | | | |  | 0.001 | | | |  |  |
| Group ✻ Sex |  | 21.948 | | |  | | 1 | | |  | | 21.948 | | |  | 0.616 | |  | 0.435 | | | |  | 0.004 | | | |  |  |
| Addition_pre |  | 2629.879 | | |  | | 1 | | |  | | 2629.879 | | |  | **73.776** | |  | **< .001** | | | |  | **0.523** | | | |  |  |
| CFT_matrices_centered |  | 4.984 | | |  | | 1 | | |  | | 4.984 | | |  | 0.140 | |  | 0.710 | | | |  | 0.001 | | | |  |  |
| Residual |  | 2352.689 | | |  | | 66 | | |  | | 35.647 | | |  |  | |  |  | | | |  |  | | | |  |  |
| **SECOND CG**  Group |  | 56.143 | | |  | | 1 | | |  | | 56.143 | | |  | 1.813 | |  | 0.183 | | | |  | 0.012 | | | |  |  |
| Sex |  | 8.713 | | |  | | 1 | | |  | | 8.713 | | |  | 0.281 | |  | 0.598 | | | |  | 0.002 | | | |  |  |
| Group ✻ Sex |  | 69.957 | | |  | | 1 | | |  | | 69.957 | | |  | 2.259 | |  | 0.138 | | | |  | 0.015 | | | |  |  |
| Addition_pre |  | 2579.806 | | |  | | 1 | | |  | | 2579.806 | | |  | **83.308** | |  | **< .001** | | | |  | **0.562** | | | |  |  |
| CFT_matrices_centered |  | 51.356 | | |  | | 1 | | |  | | 51.356 | | |  | 1.658 | |  | 0.203 | | | |  | 0.011 | | | |  |  |
| Residual |  | 1827.054 | | |  | | 59 | | |  | | 30.967 | | |  |  | |  |  | | | |  |  | | | |  |  |
| ----------------------------------------------------------------------------------------------------------------------- | | | | | | | | | | | | | | | | | | | | | | | | | | | |  |  |
| **SUBTRACTION_post** (reduced sample) – **FIRST CG**  Group |  | 38.534 | | |  | | 1 | | |  | | 38.534 | | |  | 1.386 | |  | 0.246 | | | |  | 0.022 | | | |  |  |
| Sex |  | 13.949 | | |  | | 1 | | |  | | 13.949 | | |  | 0.502 | |  | 0.483 | | | |  | 0.008 | | | |  |  |
| Group ✻ Sex |  | 0.510 | | |  | | 1 | | |  | | 0.510 | | |  | 0.018 | |  | 0.893 | | | |  | 0.000 | | | |  |  |
| Subtraction_pre |  | 522.985 | | |  | | 1 | | |  | | 522.985 | | |  | **18.809** | |  | **< .001** | | | |  | **0.292** | | | |  |  |
| CFT_matrices_centered |  | 102.286 | | |  | | 1 | | |  | | 102.286 | | |  | **3.679** | |  | **0.062** | | | |  | **0.057** | | | |  |  |
| Residual |  | 1112.202 | | |  | | 40 | | |  | | 27.805 | | |  |  | |  |  | | | |  |  | | | |  |  |
| **SECOND CG**  Group |  | 6.023 | | |  | | 1 | | |  | | 6.023 | | |  | 0.270 | |  | 0.607 | | | |  | 0.004 | | | |  |  |
| Sex |  | 3.987 | | |  | | 1 | | |  | | 3.987 | | |  | 0.179 | |  | 0.676 | | | |  | 0.003 | | | |  |  |
| Group ✻ Sex |  | 16.664 | | |  | | 1 | | |  | | 16.664 | | |  | 0.748 | |  | 0.395 | | | |  | 0.011 | | | |  |  |
| Subtraction_pre |  | 824.733 | | |  | | 1 | | |  | | 824.733 | | |  | **37.016** | |  | **< .001** | | | |  | **0.568** | | | |  |  |
| CFT_matrices_centered |  | 0.280 | | |  | | 1 | | |  | | 0.280 | | |  | 0.013 | |  | 0.912 | | | |  | 0.000 | | | |  |  |
| Residual |  | 601.576 | | |  | | 27 | | |  | | 22.281 | | |  |  | |  |  | | | |  |  | | | |  |  |

| \| Table S11  *Bayesian ANCOVA* \| \| \| \| \| \| \| \| \| \| \| \| \| --- \| --- \| --- \| --- \| --- \| --- \| --- \| --- \| --- \| --- \| --- \| --- \| \| Models \| \| P(M) \| \| P(M\|data) \| \| BF _M_ \| \| BF _01_ \| \| error % \| \| \| **FINGER GNOSIS - FIRST CG**  Null model (incl. Finger gnosis_pre1) \|  \| 0.500 \|  \| **0.797** \|  \| 3.938 \|  \| 1.000 \|  \|  \|  \| \| Group \|  \| 0.500 \|  \| 0.203 \|  \| 0.254 \|  \| **3.938** \|  \| 0.834 \|  \| \| **SECOND CG**  Null model (incl. Finger gnosis_pre) \|  \| 0.500 \|  \| **0.796** \|  \| 3.896 \|  \| 1.000 \|  \|  \|  \| \| Group \|  \| 0.500 \|  \| 0.204 \|  \| 0.257 \|  \| **3.896** \|  \| 1.252 \|  \| \| ------------------------------------------------------------------------------------------------------------------------- \| \| \| \| \| \| \| \| \| \| \|  \| \| **COMPLETION - FIRST CG**  Null model (incl. Completion_pre) \|  \| 0.500 \|  \| **0.789** \|  \| 3.736 \|  \| 1.000 \|  \|  \|  \| \| Group \|  \| 0.500 \|  \| 0.211 \|  \| 0.268 \|  \| **3.736** \|  \| 2.138 \|  \| \| **SECOND CG**  Null model (incl. Completion_pre) \|  \| 0.500 \|  \| **0.741** \|  \| 2.861 \|  \| 1.000 \|  \|  \|  \| \| Group \|  \| 0.500 \|  \| 0.259 \|  \| 0.350 \|  \| **2.861** \|  \| 0.790 \|  \| \| ------------------------------------------------------------------------------------------------------------------------- \| \| \| \| \| \| \| \| \| \| \|  \| \| **ADDITION - FIRST CG**  Null model (incl. Addition_pre) \|  \| 0.500 \|  \| **0.739** \|  \| 2.831 \|  \| 1.000 \|  \|  \|  \| \| Group \|  \| 0.500 \|  \| 0.261 \|  \| 0.353 \|  \| **2.831** \|  \| 1.149 \|  \| \| **SECOND CG**  Null model (incl. Addition_1) \|  \| 0.500 \|  \| **0.505** \|  \| 1.020 \|  \| 1.000 \|  \|  \|  \| \| Group \|  \| 0.500 \|  \| 0.495 \|  \| 0.980 \|  \| **1.020** \|  \| 1.085 \|  \| \| ------------------------------------------------------------------------------------------------------------------------- \| \| \| \| \| \| \| \| \| \| \|  \| \| **SUBTRACTION** (reduced sample)  **FIRST CG**  Null model (incl. Subtraction_1) \|  \| 0.500 \|  \| **0.644** \|  \| 1.808 \|  \| 1.000 \|  \|  \|  \| \| Group \|  \| 0.500 \|  \| 0.356 \|  \| 0.553 \|  \| **1.808** \|  \| 0.905 \|  \| \| **SECOND CG**  Null model (incl. Subtraction_1) \|  \| 0.500 \|  \| **0.716** \|  \| 2.527 \|  \| 1.000 \|  \|  \|  \| \| Group \|  \| 0.500 \|  \| 0.284 \|  \| 0.396 \|  \| **2.527** \|  \| 1.145 \|  \| |
| --- | --- | --- | --- | --- | --- | --- | --- | --- | --- | --- | --- | --- | --- | --- | --- | --- | --- | --- | --- | --- | --- | --- | --- | --- | --- | --- | --- | --- | --- | --- | --- | --- | --- | --- | --- | --- | --- | --- | --- | --- | --- | --- | --- | --- | --- | --- | --- | --- | --- | --- | --- | --- | --- | --- | --- | --- | --- | --- | --- | --- | --- | --- | --- | --- | --- | --- | --- | --- | --- | --- | --- | --- | --- | --- | --- | --- | --- | --- | --- | --- | --- | --- | --- | --- | --- | --- | --- | --- | --- | --- | --- | --- | --- | --- | --- | --- | --- | --- | --- | --- | --- | --- | --- | --- | --- | --- | --- | --- | --- | --- | --- | --- | --- | --- | --- | --- | --- | --- | --- | --- | --- | --- | --- | --- | --- | --- | --- | --- | --- | --- | --- | --- | --- | --- | --- | --- | --- | --- | --- | --- | --- | --- | --- | --- | --- | --- | --- | --- | --- | --- | --- | --- | --- | --- | --- | --- | --- | --- | --- | --- | --- | --- | --- | --- | --- | --- | --- | --- | --- | --- | --- | --- | --- | --- | --- | --- | --- | --- | --- | --- | --- | --- | --- | --- | --- | --- | --- | --- | --- | --- | --- | --- | --- | --- | --- | --- | --- | --- | --- | --- | --- | --- | --- | --- | --- | --- | --- | --- | --- | --- | --- | --- | --- | --- | --- | --- | --- | --- | --- | --- | --- | --- | --- | --- | --- | --- | --- | --- | --- | --- | --- | --- | --- | --- | --- | --- | --- | --- | --- | --- | --- | --- | --- | --- | --- | --- | --- | --- | --- | --- | --- | --- |
